# Deciphering DSC2 arrhythmogenic cardiomyopathy electrical instability: From ion channels to ECG and tailored drug therapy

**DOI:** 10.1002/ctm2.319

**Published:** 2021-02-26

**Authors:** Adrien Moreau, Jean‐Baptiste Reisqs, Helene Delanoe‐Ayari, Marion Pierre, Alexandre Janin, Antoine Deliniere, Francis Bessière, Albano C. Meli, Azzouz Charrabi, Estele Lafont, Camille Valla, Delphine Bauer, Elodie Morel, Vincent Gache, Gilles Millat, Xavier Nissan, Adele Faucherre, Chris Jopling, Sylvain Richard, Alexandre Mejat, Philippe Chevalier

**Affiliations:** ^1^ PhyMedExp INSERM U1046 CNRS UMR9214 Université de Montpellier Montpellier France; ^2^ Neuromyogene Institut Claude Bernard University, Lyon 1 Villeurbanne France; ^3^ Institut lumière matière Claude Bernard University, Lyon 1 Villeurbanne France; ^4^ Service de Rythmologie Hospices Civils de Lyon Lyon France; ^5^ Laboratoire de Cardiogénétique moléculaire Centre de biologie et pathologie Est Bron France; ^6^ CECS I‐Stem Corbeil‐Essonnes France; ^7^ IGF, CNRS, INSERM Université de Montpellier Montpellier France

**Keywords:** action potential duration, arrhythmogenic cardiomyopathy, desmocollin, hiPSC‐CM, QT duration

## Abstract

**Background:**

Severe ventricular rhythm disturbances are the hallmark of arrhythmogenic cardiomyopathy (ACM), and are often explained by structural conduction abnormalities. However, comprehensive investigations of ACM cell electrical instability are lacking. This study aimed to elucidate early electrical myogenic signature of ACM.

**Methods:**

We investigated a 41‐year‐old ACM patient with a missense mutation (c.394C>T) in the *DSC2* gene, which encodes desmocollin 2. Pathogenicity of this variant was confirmed using a zebrafish *DSC2* model system. Control and *DSC2* patient‐derived pluripotent stem cells were reprogrammed and differentiated into cardiomyocytes (hiPSC‐CM) to examine the specific electromechanical phenotype and its modulation by antiarrhythmic drugs (AADs). Samples of the patient's heart and hiPSC‐CM were examined to identify molecular and cellular alterations.

**Results:**

A shortened action potential duration was associated with reduced Ca^2+^ current density and increased K^+^ current density. This finding led to the elucidation of previously unknown abnormal repolarization dynamics in ACM patients. Moreover, the Ca^2+^ mobilised during transients was decreased, and the Ca^2+^ sparks frequency was increased. AAD testing revealed the following: (1) flecainide normalised Ca^2+^ transients and significantly decreased Ca^2+^ spark occurrence and (2) sotalol significantly lengthened the action potential and normalised the cells’ contractile properties.

**Conclusions:**

Thorough analysis of hiPSC‐CM derived from the *DSC2* patient revealed abnormal repolarization dynamics, prompting the discovery of a short QT interval in some ACM patients. Overall, these results confirm a myogenic origin of ACM electrical instability and provide a rationale for prescribing class 1 and 3 AADs in ACM patients with increased ventricular repolarization reserve.

## Graphical abstract

Thorough analysis of hiPSC‐CM derived from a DSC2 patient, zebrafish and patient cohort, we identified abnormal repolarization dynamicity, prompting the discovery of a short QT interval in some ACM patients. By normalizing the increased repolarization reserve of ACM myocytes, class 3 AADs are likely to be the drugs of first choice for DSC2 patients. These findings may encourage randomized trials to evaluate class 3 antiarrhythmic drugs, alone or in combination with class I medications in ACM patients.

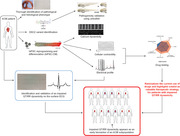

## 1 INTRODUCTION

Arrhythmogenic cardiomyopathy (ACM) refers to a group of rare hereditary cardiac diseases characterised by lethal ventricular arrhythmias (VA) and progressive fibro‐fatty replacement of myocardial tissue. Its prevalence is estimated at one in 2000–5000.^1^, ^2^, ^3^, ^4^ The disease develops in three stages: (1) a concealed phase, with no mechanical dysfunction but a risk of sudden cardiac death, followed by (2) myocardial structural alteration and (3) progression towards heart failure.^3^, ^5^ Mutations in genes coding for desmosomal proteins are common in the right ventricular form of ACM disease.^3^, ^6^ *PKP2* mutations are the most common, whereas *DSC2* are among the rarest (1%–2%).^3^ Gehmlich et al. identified two missense mutations of *DSC2* gene (R203C and T275 M) that showed defections in proteolytic cleavage, a process that is essential for activated cadherins.^7^ Although *DSC2* variants are rare in patients with ACM, their pathogenic role has been consistently demonstrated. A case report by Christensen and co‐workers have suggested a functional role of *DSC2* c.1445G>C in a 31‐year‐old White male with ACM complicated with ventricular fibrillation.^8^ Knockdown of the DSC orthologue in zebrafish caused a cardiomyopathy phenotype.^9^ In a transgenic mice model with cardiac specific overexpression of DSC2, severe myocardial necrosis, fibrosis and inflammation mimicking ACM were found.^10^

Desmosomes are structures located at intercalated discs in the myocardium tissue. They are responsible for intercellular adhesion and composed of three main protein families: cadherins (desmocollins (*DSC*) and desmogleins (*DSG*)), armadillo proteins (junction plakoglobin (*JUP*) and plakophilins (*PKP*)) and plakins (desmoplakin (*DSP*)).^1^, ^11^, ^12^, ^13^, ^14^ Desmocollins (*DSC*) and desmogleins (*DSG*) mediate cell–cell interaction through their extracellular domain. Their intracellular domain is connected to cytoskeleton proteins through adaptors, which are junction plakoglobin (*JUP*), plakophilin‐2 (*PKP2*) and terminally desmoplakin (*DSP*). This desmosomal protein complex consequently ensures solid intercellular junctions and notably explains why these structures are mainly found in stretched tissues such as the skin or the heart.^1^, ^15^, ^16^, ^17^ Desmosomes were initially described to have a specific physical role of intercellular adhesion, linking intracellular cytoskeleton to extracellular cadherins domain. Desmosome physical destabilization leading to cardiomyocyte death and fibro‐fatty replacement constituted the first proposed pathomechanism of ACM.^11^, ^14^, ^17^ A parallel hypothesis underlying the fibro‐fatty inclusion in ACM comes from the potential trans‐differentiation of cardiomyocytes into adipocytes.^18^

The ACM pathogenesis is not fully understood. Prescription of antiarrhythmic drugs (AADs) remains partly empirical and automatic defibrillators are often implanted. Human‐induced pluripotent stem cells (hiPSCs) can be used to elucidate disease mechanisms.^19^, ^20^, ^21^ Studies using cardiomyocytes derived from ACM patient‐specific hiPSCs (hiPSC‐CM) are expected to enable a better understanding of arrhythmogenesis.^22^, ^23^ Here, a thorough functional description of hiPSC‐CM from a *DSC2* ACM patient revealed primary electrical abnormalities and responsiveness to AADs. Of note, we found that a short QT interval at low heart rate is characteristic of *DSC2* ACM.

## 2 METHODS

The local ethics committee approved the study protocol (DC‐2008‐72/DC‐2019‐3464). The study was conducted according to the principle of the declaration of Helsinki. Informed consents were obtained for all cases.

### 2.1 Biological samples

Biological samples were collected from the ACM patient or the control after cardiac transplantation. The explanted heart of the ACM patient was subjected to macroscopic and histopathological examinations. Myocardial samples were collected from the anterior and posterior left and right ventricular walls. Specimens were either formalin fixed and paraffin embedded for histological investigation or snap frozen in liquid nitrogen and stored at –80°C until RNA analysis. RNA extraction was performed as described below.

Histological sections for light microscopy were stained with the haematoxylin–eosin–safran (HES) technique.

### 2.2 Patient cohort analysis

The study was retrospective and involved 79 patients (nine women and 70 men) with an average age of 36 years at the time of their first documented ventricular tachycardia (VT) and admitted to hospital between 1971 and 2016. The patients were diagnosed using Task Force criteria.^24^ Fifty‐nine patients were treated by AADs alone; 17 by AADs and tachycardia ablation. An automatic defibrillator was implanted in five patients. Familial forms of ACM were observed in 38% (including diverse genetic profiles). Forty‐one patients had sustained VT and 18 patients had nonsustained VT. The abnormalities of the right ventricular wall motion on ventriculography were localised in 45% of cases and diffused in 55% of cases.

### 2.3 Genetic analysis

Blood samples from the ACM patient were subjected to direct sequencing to investigate six ACM‐related genes (*RYR2*, *PKP2*, *DSG2*, *DSC2*, *DSP*, *CASQ2* and *JUP*). Genomic DNA was extracted from leucocytes present in whole blood samples with the classical phenol chloroform technique. Each exon and all intron–exon junctions for the studied genes were amplified with primers designed with PRIMER3 software (characteristics of primers available upon request). The entire coding sequences were analysed for the following genes: *JUP* (Online Mendelian Inheritance in Man identifiers: **OMIM 173325**, **MN_021991.2**, transcript 745aa), *DSP* (**OMIM 125647**, **MN_004415.2**, transcript 2871aa), *PKP2* (**OMIM 602861**, **MN_004572.3**, transcript 881aa), *DSG2* (**OMIM 125671, MN_001943.3**, transcript 1118aa) and *DSC2* (**OMIM 125645**, **MN_024422.3**, transcript 901aa). Direct sequencing was performed with BYG DYE dideoxy‐terminator chemistry (Perkin Elmer) on an ABI 3830 DNA sequencer (PE Applied Biosystems). Analysis of the chromatograms was performed with SeqScape (PE Applied Biosystems). Analysis of all five genes was performed in all samples, even when a mutation was identified in a given gene. A control group of 300 healthy unrelated subjects (600 alleles) of Caucasian origin was also genetically profiled for comparison.^25^

### 2.4 Culture and differentiation of hiPSC

Biological samples were handled in accordance with the Declaration of Helsinki with informed consent and standardised approved protocols by a local ethic committee.

Both controls hiPSC lines were a kind gift from (1) the platform iPS_INMG (hiPSC AG08C5; neuromyogen institute, Lyon)^26^ and from (2) Xavier Nissan (hiPSC Fsc03; I‐Stem institute, Evry). Both control hiPSC lines were derived from males. Patient‐specific hiPSC were reprogrammed and characterised at Phenocell (Evry, France) core facility using the episomal transfection of five reprogramming factors: Oct4, Sox2, Klf4, L‐Myc and Lin28. The reprogramming process was performed on skin fibroblasts, in feeder‐free condition with the STEMACS IPS Brew‐XF medium. All hiPS cells used underwent a standard validation procedure notably including the validation of pluripotency markers and differentiation potential.

The hiPSC were grown on an hESC‐qualified Matrigel™ (Corning) and adapted to StemFlex™ medium (ThermoFisher Scientific) and were passaged using TrypLE™ Express (ThermoFisher Scientific). The published sandwich monolayer technique was adapted to differentiate hiPSC.^19^, ^20^, ^27^ In brief, hiPSC were dissociated with TrypLE™ Express and 30,000–50,000 cells/cm^2^ were plated on hESC‐qualified Matrigel in StemFlex™ medium for 4 days with daily media replacement. The next day, the medium was replaced by StemFlex™ medium supplemented with Matrigel growth factor reduced (MGFR; 0.04 mg of Matrigel protein per millilitre of medium). The differentiation process was then induced with RPMI 1640 medium supplemented with B27 supplement minus insulin (1×), 6 μM CHIR99021 (LC Laboratories) and MGFR (0.04 mg of Matrigel protein per millilitre of medium) for 2 days. On day 2, the medium was replaced by RPMI 1640 supplemented with B27 supplement minus insulin (1×). On days 3 and 5, the medium was replaced by RPMI 1640 supplemented with B27 supplement minus insulin (1×). At day 3 specifically, the medium was supplemented with 2 μM of Wnt–C59 (LC Laboratories). At days 7 and 9, the medium was replaced with RPMI 1640 supplemented with B27 supplement minus insulin (1×). The medium was renewed three times a week with RPMI 1640 supplemented with B27 supplement (1×). The differentiation process resulted in spontaneously beating cells observed from day 7. When required, after 23 or 53 days of differentiation, cells were enzymatically dispersed using TrypLE™ Express. When required, cells were frozen using a standard protocol. The freezing medium consisted 50% of RPMI 1640 with B27 supplement, 40% of foetal bovine serum and 10% of dimethyl sulfoxide.

### 2.5 Reverse transcription

Total RNA was extracted from hiPSC‐CM (D60 of differentiation) using Direct‐zol™ RNA MiniPrep Kit (Zymo Research, Ozyme) according to the manufacturer's instructions. Total RNA was quantified using NanoDrop 1000 spectrophotometer (Thermo Fisher Scientific). Based on 500 ng of total RNA extracted from the hiPSC‐CM, reverse transcription was performed using the GoScript Reverse Transcription system (Promega) according to the manufacturer's protocol.

### 2.6 Reverse transcription and quantitative reverse transcription polymerase chain reaction analysis

hiPSC‐CM cDNA were measured by quantitative reverse transcription polymerase chain reaction (qRT‐PCR) with the SYBR Green PCR kit for LightCycler (Roche Diagnostics, Indianapolis, IN) according to manufacturer's recommendations. The Rotor‐Gene Q real‐time PCR Cycler l run protocol consisted of initial Taq activation at 95°C for 10 min followed by 39 cycles of 95°C for 10 s, 60°C for 30 s and fluorescence reading at each cycle end. Finally, Taq is inactivated at 65°C for 30s. To control for amplification variations due to differences in the starting mRNA concentrations, we used the ribosomal protein L22 (*RPL22*) mRNA as an internal control. The mRNA levels for each tissue were normalised to the *RPL22* mRNA levels. The quantifications were computed from the threshold cycle values obtained for the gene of interest and the efficiency of the primer set with Rotor‐Gene^®^ Q software.

Heart samples (patient or control) synthesised cDNA was measured by qRT‐PCR with the SYBR Green PCR kit for LightCycler (Roche Diagnostics, Indianapolis, IN) according to manufacturer's recommendations. LightCycler experimental run protocol consisted of initial Taq activation at 95°C for 8 min and 45 cycles of 95°C for 15 s, 68°C for 5 s and 72°C for 7 s. Quantification was performed with a single fluorescence measurement. To control for amplification variations due to differences in the starting mRNA concentrations, we used the ribosomal protein L21 (*RPL4*) mRNA as an internal control. The mRNA levels for each tissue were evaluated relative to the *RPL4* mRNA levels. The quantifications were computed from the threshold cycle values obtained for the gene of interest and the efficiency of the primer set with RealQuant software (Roche Diagnostics).

Healthy donor's cardiac tissue was obtained from a 48‐year‐old patient without any family history of cardiac disease. It was used as a reference to compare mRNA expression with patient's RV.

### 2.7 Immunostaining

#### 2.7.1 Heart sections

Cardiac segments embedded in paraffin blocks were cut into 4 μm slides. The Ventana Roche automated immunohistochemistry stainer (Discovery XT) was used. Detection of immunoreactivity was performed with the Ventana Ultraview DAB Kit. Antigen retrieval was performed in citrate buffer. Sections were incubated with antibody anti‐plakoglobin (i.e. ã‐catenin) (rabbit polyclonal, Cell signaling technology, #2309). Haematoxylin and bluing reagent were used for counterstaining.

#### 2.7.2 hiPSC‐CM immunolabelling

Seven days prior to experiments, hiPSC‐CM cells were dissociated and plated onto glass coverslips. The cells were fixed using 37°C PBS – 4% paraformaldehyde – 4% sucrose solution for 20 min. Cells were then permeabilised for 30 min at room temperature using 0.1% triton in a PBS – 1% bovine serum albumin solution. Primary anti‐bodies to detect myosin–light chain–2v (mlc2v, 1:300, AP02680PU–N, Acris antibodies), desmocolin 2 (Progen 610120)) were incubated overnight at 4°C. Fluorophore‐conjugated secondary anti‐bodies (Alexa Fluor^®^ 488 goat anti rabbit (1:250, A11070, Life Technologies)) were incubated for 1 h at room temperature. The nucleus was labelled using DAPI (4′,6–diamidino‐2‐phenylindole dihydrochloride). Cells were observed on a Zeiss LSM confocal microscope with a 63× oil objective equipped with appropriate laser and filters.

Images of the mlc2v protein obtained with the confocal microscope were extracted and converted into 8‐bit grey scale. Images were then studied using a homemade Matlab^®^ analysis script to evaluate sarcomeres characteristics (cell area covered by a labelling, organization into doublets, sarcomeres orientation).

### 2.8 Western blot

Control and patient heart samples were snap frozen in liquid nitrogen. Proteins from sample lysate were then isolated. Equal amounts of total proteins (50 μg) were denatured in Laemmli buffer (Sigma), resolved on 10% SDS–polyacrylamide gel and blotted onto 0.45 μm Immobilon polyvinylidene difluoride membrane. Membranes were blocked and then incubated with primary antibody (Rabbit‐anti‐DSC2, Progen 610120) followed by appropriate anti‐rabbit horseradish peroxidase–conjugated secondary antibodies.

### 2.9 hiPSC‐CM contraction analysis

The hiPSC‐CM contractile function was assessed through a patented custom‐made video analysis. For this purpose, hiPSC‐CM monolayers (without any cellular dissociation) in six‐well dishes were placed on the stage of an inverted microscope equipped with a 10× objective and an incubation chamber. The temperature and CO_2_ levels were set as follow: 37°C, 5% (humid atmosphere). Cells were allowed to stabilise for at least 15 min prior to any recordings. Phase contrast videos were acquired at 30 frames per second. Videos were then processed: TIFF images were extracted, and contrasted particles displacement was tracked frame by frame for each video. The displacement of each contrasted particles was then processed through the time resulting in a curve of the displacement as a function of time. Areas with similar contractile behaviour are clustered and contractile parameters quantified.

### 2.10 Electrophysiology

Patch clamp experiments were performed at room temperature using an Axopatch 200B amplifier (Axon Instruments, Foster City, CA, USA) at least 5 days after dispersion of the hiPSC‐CM (using TrypLE express^®^). For all experiments, the liquid junction potential between the patch pipette and the bath solution was not corrected. Pipettes were made from borosilicate glass capillaries and fire‐polished. For voltage‐gated sodium (Na^+^) channels recordings (voltage‐clamp experiments), pipettes were coated with HIPEC (Dow–Corning, Midland, MI, USA) to minimise electrode capacitance.

Action potentials (APs) were evaluated using the whole cell configuration (in current clamp mode). The gap‐free mode was used to record spontaneous APs. For this purpose, the electrical activity was recorded at basal state. For other current clamp experiments, APs were elicited following injection of a 3 ms, 20‐1500 pA rectangular current pulse at different rates (0.5, 1 and 2 Hz). The patch pipettes (resistance 2–5 mOhm) were filled with a solution containing (in mmol/L): 10 NaCl, 122 KCl, 1 MgCl_2_, 1 EGTA and 10 Hepes; pH was adjusted at 7.3 with KOH. The bath solution (external current clamp) was composed of (in mmol/L): 154 NaCl, 5.6 KCl, 2 CaCl_2_, 1 MgCl_2_, 8 Glucose and 10 Hepes; the pH was adjusted at 7.3 with 1 N NaOH.

Macroscopic Na^+^ currents were recorded using the whole‐cell configuration but under voltage‐clamp conditions. The patch pipettes (resistance 1–2 mOhm) were filled with a solution containing (in mmol/L): 105 CsF, 35 NaCl, 10 EGTA and 10 Hepes; pH was adjusted to 7.4 using 1 N CsOH. The bath solution was composed of (in mmol/L): 105 NMDG, 35 NaCl, 2 KCl, 1.5 CaCl_2_, 1 MgCl_2_, 10 glucose, 10 Hepes, 10 TEA‐Cl and 0.5 Nimodipine; pH was adjusted to 7.4 using methanethiosulfonic acid. Voltage‐clamp command pulses were generated by a microcomputer using pCLAMP software v10 (Axon Instruments). Na^+^ currents were filtered at 5 kHz, digitised at 10 kHz and stored on a microcomputer equipped with an AD converter (Digidata 1440A, Axon Instruments). P/4 leak subtraction was used prior to test pulses.

For macroscopic voltage‐gated calcium (Ca^2+^) and potassium (K^+^) channels recordings, similar experimental condition was used but with no P/4 leak subtraction. For Ca^2+^ channels currents, the patch pipettes were filled with a solution containing (in mmol/L): 35 CsCl, 100 CsF, 5 Na_2_ATP, 10 EGTA and 10 HEPES; pH was adjusted to 7.4 using 1 M KOH. The bath solution consisted (in mmol/L): 140 TEA‐Cl, 2 KCl, 2.5 BaCl_2_, 2 MgCl_2_, 10 glucose and 10 HEPES; pH was adjusted to 7.4 using 1 M KOH.

For K^+^ currents, the patch pipets were filled with a solution containing (in mmol/L): 10 NaCl, 122 KCl, 1 MgCl_2_, 1 EGTA and 10 Hepes; pH was adjusted at 7.3 with KOH. The bath solution (external current clamp) was composed of (in mmol/L): 154 NaCl, 5.6 KCl, 2 CaCl_2_, 1 MgCl_2_, 8 Glucose and 10 Hepes; the pH was adjusted at 7.3 with 1 N NaOH.

### 2.11 Calcium handling studies

For Ca^2+^ transient measurements, hiPSC‐CMs were loaded with 3 μM Fluo‐4 AM Ca^2+^ indicator (Molecular Probes, Em/Ex: 494/506 nm) incubated in the hiPSC‐CM culture medium (RPMI 1640 + B27 1×) for 20 min. Recordings were performed in a physiological solution (154 mM NaCl; 5.6 mM KCl; 2 mM CaCl_2_; 1 mM MgCl_2_; 8 mM Glucose; 10 mM HEPES). Ca^2+^ images were recorded with an inverted confocal microscope (Zeiss LSM 810) equipped with a 63× objective (oil immersion, numerical aperture, N.A. = 1.4). Confocal images were obtained in line scan mode (i.e. *x*–*t* mode, 1.24 ms per line; 512 pixels × 5000 lines). To enable comparisons between cells, changes in the Fluo‐4 fluorescence signal (Δ*F*) were normalised by basal fluorescence (*F*_0_). All data were extracted using AIM 4.2 (Zeiss). Maximal amplitudes and event frequencies were extracted and analysed from raw data by in‐house developed algorithm implemented in Python (version 3.0) (https://asalykin.github.io/PeakInspector/) and Prism (GraphPad). Sparks events were analysed using the imageJ plug‐in SparkMaster (https://sites.google.com/site/sparkmasterhome/).

### 2.12 Zebrafish strains and husbandry: Morpholinos and injections

Zebrafish were maintained under standardised conditions and experiments were conducted in accordance with the European Communities council directive 2010/63.

Morpholino oligonucleotides (MOs) were obtained from Gene Tools (Philomath, OR, USA) and injected into SAT wild‐type one‐cell stage embryos.

The dsc2l ATG‐MO morpholino targets the start codon of dsc2l. The dsc2l splice‐MO2 targets the exon2/intron2 splice site of dsc2l.

The sequences of the injected MOs are the following: dsc2l ATG‐MO – 5′‐GAGTTCTCATGCCATTGAAGTCTAC‐3′ (10 ng); dsc2l splice‐MO – 5′‐AACCAAACATTCTTCCGCACCTTGA‐3′ (5 ng).

For rescue experiments, total mRNAs were extracted from human iPS‐derived cardiomyocytes using Trizol^®^. *DSC2* cDNAs were cloned into pGem‐T Easy (Invitrogen) and human *DSC2* mRNAs were synthesised using mMessage mMachine (Ambion).

### 2.13 Zebrafish's cardiovascular parameters analysis

To determine cardiovascular parameters, we used μZebraLab™ software from ViewPoint, which has been developed specifically for this purpose. All experiments were performed as described previously.^28^ To determine the mean blood flow, the average of the blood flow values was calculated from a 30‐s time frame (3900 frames). Stroke volume was calculated by dividing the average blood flow (nL/s) by the heart beat per second (BPM/60). To assess heart contractility, movies were recorded at 120 fps and measurements were made using ImageJ software. Data were collected from three different experiments (four to 12 embryos per experiments).

### 2.14 Data analysis and statistics

The electrophysiological results were analysed using Clampfit (pCLAMP v10.0; Molecular Devices) and custom‐written MATLAB programs (The MathWorks Inc.). The contractile function was analysed using custom‐written MATLAB programs. The confocal microscopic results were analysed using ImageJ and custom‐written MATLAB programs.

A normality test (Agostino and Pearson omnibus normality test) was always used to determine whether data follow a normal distribution. In most of cases, the normality test failed indicating a nonnormal distribution. In such cases, data are expressed as median (lower 95% confidence interval/upper 95% confidence interval). When specifically stated, results are expressed as means ± SEM. When indicated, for comparing two conditions, a parametric or nonparametric *t*‐test was performed using GraphPad prism software (GraphPad Software, Inc.). When indicated, for comparing more than two conditions with nonnormal distribution, a nonparametric Kruskal–Wallis test (with Dunn's correction for multiple comparisons) was performed using GraphPad prism software. Differences were considered significant at *p* < 0.05 (*), *p* < 0.01 (**), *p* < 0.001 (***) or *p* < 0.0001 (****).

## 3 RESULTS

### 3.1 Patient history

**FIGURE 1:**
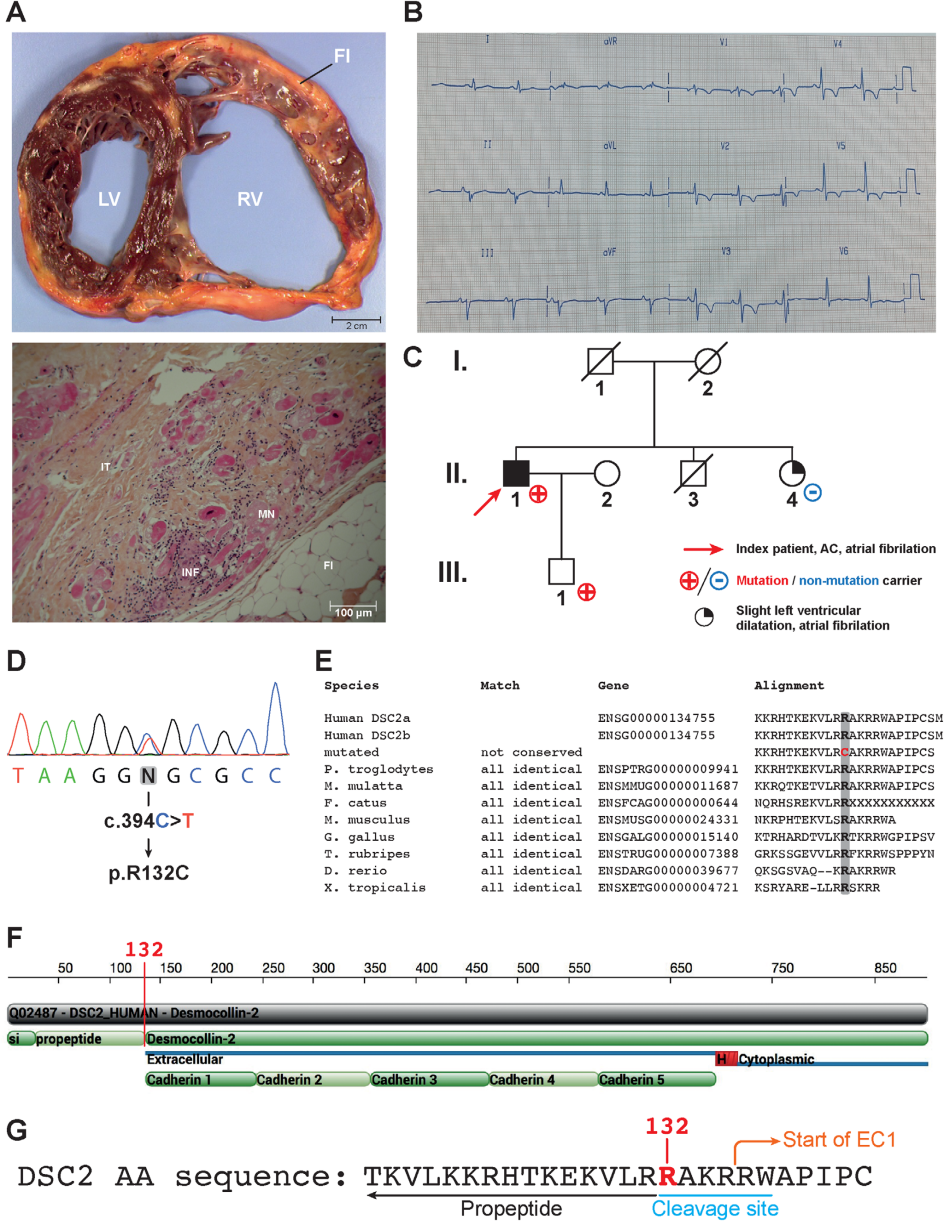
Clinical presentation. (A) Upper panel: transverse section of the ACM patient's explanted heart. The right ventricle (RV) is dilated. Adipo–fibrotic replacement of the myocardium is also observed. Lower panel: Microscopic section of the patient's explanted heart stained with hematoxylin, eosin and safran. Inflammation, adipose tissue and myocardial necrosis are observed. IT, interstitial tissue; INF, inflammation; FI, adipose tissue; MN, myocardial necrosis. (B) Twelve‐lead surface ECG illustrating the patient's electrical profile. The patient was in sinus rhythm with a PR interval at 160 ms, QRS interval at 120 ms and S wave slurring. Negative T waves were observed in V2 through V5. (C) Family pedigree. The index patient (II.1) is indicated by a red arrow. Individuals indicated with a red cross carry the mutation (II‐1 and III‐1). (D) Electropherogram of the patient's DNA showing the *DSC2* mutation responsible for the R132C substitution. (E) Multispecies protein sequence alignment illustrating the high degree of conservation of the arginine at position 132 (highlighted in grey). (F) Two‐dimensional schematic of the DSC2 protein sequence showing the propeptide and cleavage site. Arginine 132 is located at the junction of the propeptide and the mature protein. (G) DSC2 protein sequence illustrating the location of arginine 132 within the cleavage site. Cleavage activates desmocollin's adhesive properties

Heart samples were obtained from two Caucasian patients. A control heart was obtained from a 48‐year‐old patient with no family history of cardiac disease. This heart was initially meant for heart transplant, but due to technical issue, transplant was cancelled. A diseased heart was obtained from the index patient with ACM diagnosed at 41 years of age (Figure 1). This patient had been resuscitated from sudden cardiac death after an episode of VT, leading to the implantation of a cardiac defibrillator at age 50. The right ventricle (RV) ejection fraction was severely depressed. The patient suffered from various ventricular arrhythmias such as premature ventricular complexes. The ECG recapitulated many criterions from the ACM task force criteria.^24^ In this example, the patient was in sinus rhythm with a PR interval at 160 ms, QRS interval at 120 ms and S wave slurring. Negative T waves were observed in V2 through V5 (Figure 1B). The patient underwent heart transplantation at age 66. The family history was negative for cardiomyopathy, but the patient's only son refused to be clinically tested (Figure 1C). Macroscopic observation of the ACM patient's heart showed an enlargement of the RV (Figure 1A, upper panel). The myocardial layers were replaced by fibro‐fatty tissue, mainly in the RV (Figure 1A). There were abundant lymphocytes but no neutrophils in both the subendocardium and subepicardium. Extensive mutilating fibrosis and myocardial necrosis were also present (Figure 1A, bottom panel).

A heterozygous missense mutation (c.394C>T) in exon 3 of the *DSC2* gene (NM_024422.6:c.394C>T[p.Arg132Cys], genomic location NC_000018.10:g.31091108G>A) (Figures 1C and 1D) coding for amino acid replacement of arginine by cysteine at position 132 was identified. This variant affected the *DSC2a* and *DSC2b* genes (Figure 1D–G). Its allele frequency was estimated at 3.3 × 10^–5^ in the ExAC database and 3.5 × 10‐5 in gnomAD database (single‐nucleotide variant: 18‐28671071‐G‐A). This variant was identified in a Japanese proband with ACM diagnosed at 15 years of age.^29^ No mutations were found in the *RYR2*, *PKP2*, *DSG2*, *DSP*, JUP or *CASQ2* genes in the patient in our study.

### 3.2 The DSC2/R132C substitution affects zebrafish heart function

**FIGURE 2:**
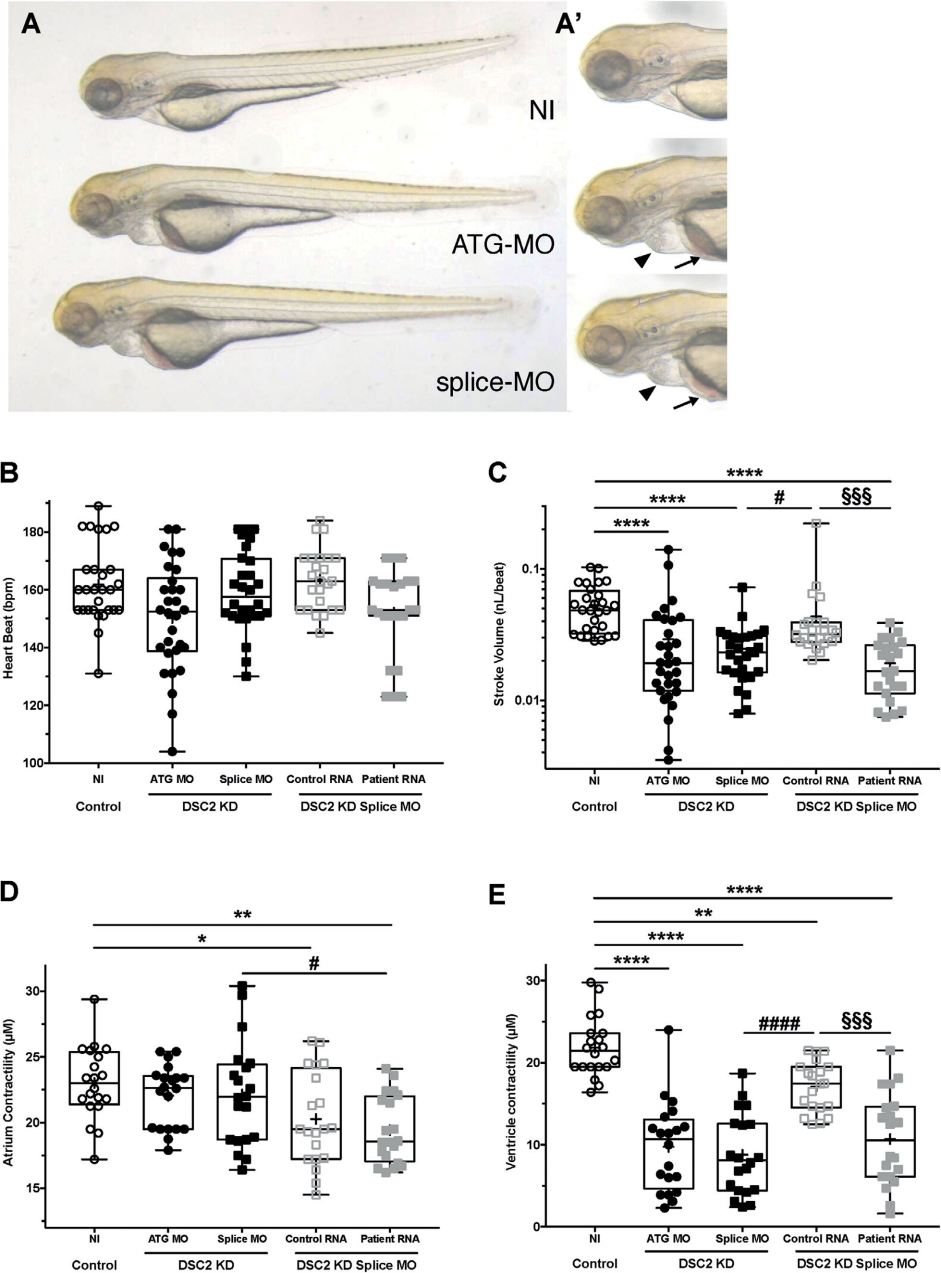
In vivo effect of DSC2 knockdown and mutation. (A) Bright‐field images of noninjected (NI), *dsc2l* ATG‐ and splice‐MO‐injected fish. (A') The same larvae at higher magnification. Both morphants display cardiac oedema (black arrowheads) and blood accumulation (black arrows). (B) Box plot depicting the average heart rate in beats per minute of non‐injected (NI, *N* = 28) and *dsc2l* ATG‐ (*N* = 30), splice‐morphant (*N* = 28) larvae and larvae co‐injected with dsc2l splice MO and human *DSC2* wild‐type mRNA (*N* = 24) or mutated mRNA (*N* = 23). (C) Box plot depicting the average stroke volume in nanolitres per beat of non‐injected (NI, *n* = 28), *dsc2l* ATG‐ (*N* = 30), splice‐morphants (*N* = 28) and larvae co‐injected with dsc2l splice MO and human *DSC2* wild‐type mRNA (*N* = 24) or mutated mRNA (*N* = 23). (D) Box plot depicting the average atrial contractile distance in micrometres (μm) of non‐injected (NI, *N* = 22) and *dsc2l* ATG‐ (*N* = 20) and splice‐morphants (*N* = 20), and larvae co‐injected with *dsc2l* splice‐MO and human *DSC2* wild‐type mRNA (*N* = 20) or mutated mRNA (*N* = 20). (E) Box plot depicting the average ventricular contractile distance in micrometres (μm) of non‐injected (NI, *N* = 22), *dsc2l* ATG‐ (*N* = 20), splice‐morphants (*N* = 20) and larvae co‐injected with *dsc2l* splice‐MO and human *DSC2* wild‐type mRNA (*N* = 20) or mutated mRNA (*N* = 20). *p < 0.05, **p < 0.01, ***p < 0.001, and ****p < 0.0001 (Kruskal–Wallis test). “*” indicates a value significantly different from that for the non‐injected, “#” indicates a value significantly different from that for the *dsc2l* splice‐MO, and “§” indicates a value significantly different from that for the larvae co‐injected with *dsc2l* splice‐MO and human *DSC2* wild‐type mRNA

We used the zebrafish model to validate the effect of the DSC2 p.R132C substitution *in‐vivo*. Two morpholinos were designed against the *DSC2* zebrafish orthologue *dsc2l* (ENSDARG00000039677), targeting either the start codon (ATG‐MO) or the exon2/intron2 splice site of *dsc2l* (splice‐MO). At 3 days post‐fertilization, ATG‐MO morphant larvae showed cardiac oedema associated with blood accumulation around the yolk sac, as previously described.^9^ The same phenotype was observed in embryos that received the splice‐MO (Figures 2A and 2A').

We analysed several physiological parameters using the ViewPoint ZebraBlood system. The heart rate was not affected when *dscl2l* was knocked down (Figure 2B; Table S1). However, both ATG and splice *dsc2l* morphants exhibited decreased stroke volumes (Figure 2C; Table S1). Because human patients with *DSC2* mutations harbour defects in RV contractility, we analysed and compared high‐speed video recordings of wild‐type un‐injected embryos and *dcsc2l* morphants. Ventricular contraction was significantly decreased in both ATG and splice morphants compared with wild‐type embryos, similar to a previously published report (Figures 2D and 2E; Table S1 and Movies S1–S3).^9^

By adopting the same strategy as Heusser et al., we sought to determine whether the R132C substitution affected DCS2 protein function. First, we attempted to rescue the morphant phenotype using full‐length human *DCS2* mRNA. Co‐injection of the *dsc2l* splice‐MO with 100 pg of control *DCS2* mRNA significantly rescued the stroke volume and ventricle defects observed in *dsc2l* morphants (Figure 2C–E; Table S1). We performed the same experiment using the mutated human mRNA encoding the R132C substitution. Unlike the wild‐type mRNA, the mutated mRNA failed to rescue the cardiovascular phenotype, indicating that the R132C substitution impairs DSC2 function *in‐vivo* (Figure 2C–E; Table S1).

### 3.3 Molecular and cellular impairment of ACM heart and hiPSC‐CM

From heart samples, when compared with mRNA from the control RV, that from the patient's explanted RV revealed down‐regulation of desmosomal genes including *DSC2*, *DSG2*, *PKP2*, *DSP* and *JUP* (Figure S1A–E). The percentage difference in the *DSC2* and *JUP* mRNA levels measured in control and patient's hiPSC‐CM was highly similar to that quantified in the control versus patient heart samples (Figure S1F). Immunolabelling of control and patient heart samples showed that the aligned‐fibre organization was partially lost in the patient heart (Figures S1G and S1H). Plakoglobin (JUP) immunostaining was reduced in the patient heart sample (Figure S1H). Consistent with lower *DSC2* mRNA levels in both the patient heart and the patient‐derived hiPSC‐CM, western blot experiments from patient explanted heart samples confirmed the lower DSC2 protein expression level (Figure S1I). Furthermore, in patient's hiPSC‐CM immunostaining showed decreased DSC2 labelling and a loss of the membrane enrichment when compared to control hiPSC‐CM (Figure S1J). In hiPSC‐CM, sarcomeric organization was affected, with a partial loss of sarcomere formation (Figure S1K). The fractional area staining positive for ventricular myosin light chain 2 (MLC2v) was reduced in the patient hiPSC‐CM relative to the control (Figures S2A and S2B). The patient hiPSC‐CM also demonstrated a lower level of striation when compared with the control hiPSC‐CM (Figure S2C–E), consistent with the decreased organization observed in the patient's heart sections.

### 3.4 DSC2 patient hiPSC‐CM demonstrate altered contractile properties

**FIGURE 3:**
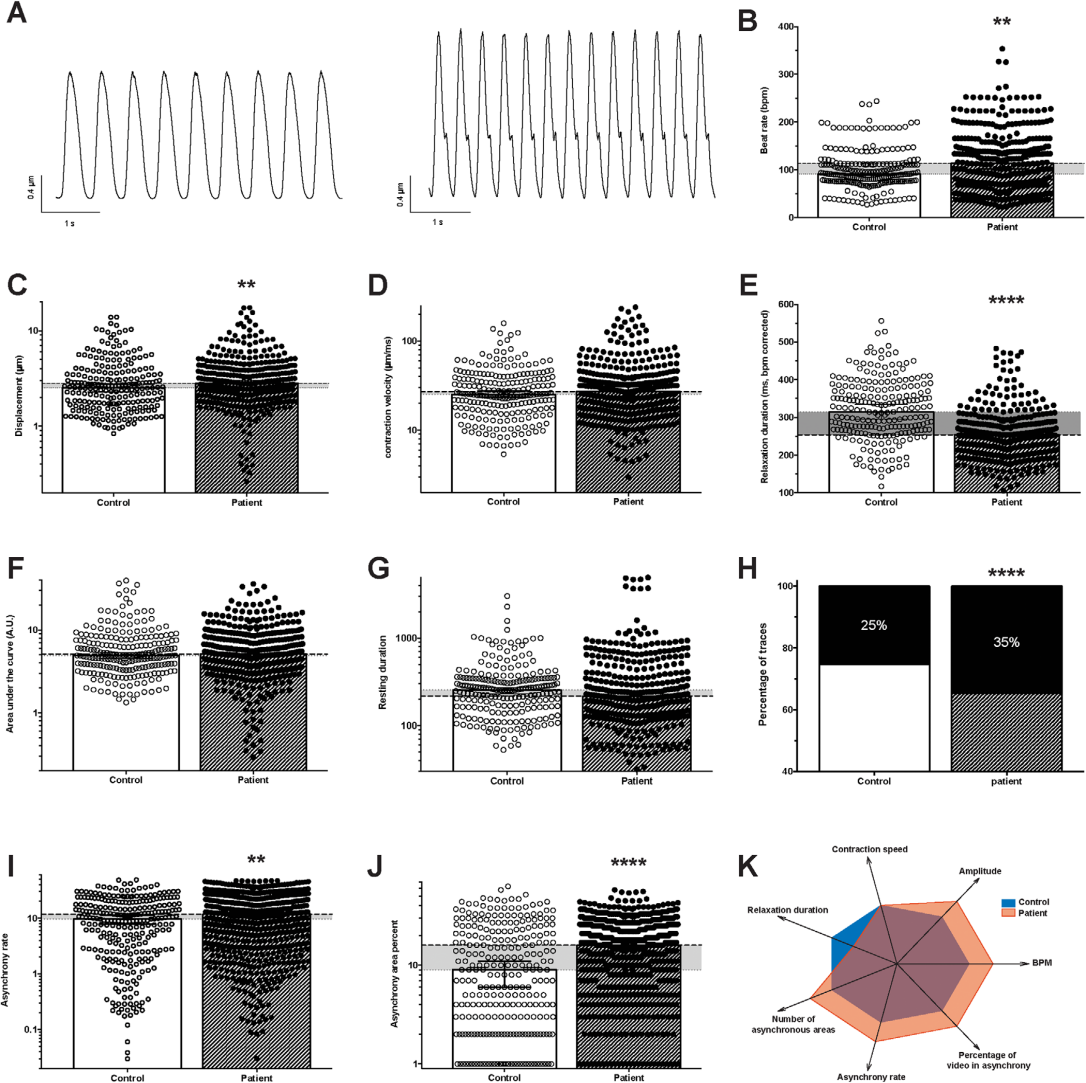
Evaluation of control and patient‐specific hiPSC‐CM spontaneous contractile function by video microscopy. (A) Contraction cycles from phase‐contrast videos of control (left) and patient‐specific (right) hiPSC‐CM monolayers obtained using custom made software analysis. (B–J) Contraction characteristics of control (*N* = 229) and patient‐specific monolayers (*N* = 359). (B) Beat rate, (C) cellular displacement, (D) contraction velocity, (E) contraction duration from peak to 90% relaxation corrected with Bazett's formula, (F) area under the curve, (G) resting duration, (H) the percentage of videos demonstrating aberrant contractile events (control: 25% (278 areas out of 1097 total areas); patient specific: 35% (600 areas out of 1727 total areas)), (I) the percentage of recording spent in asynchrony and (J) the percentage of the video area concerned by aberrant events. (K) Spider chart illustrating the main differences observed between control (blue area) and patient‐specific (orange area) hiPSC‐CM. A “normal” value of 2 is attributed to the control parameter. Each evaluated parameter is compared to the control condition and fixed as higher (3) or lower (1) if statistically significantly different. Histograms represent the median (95% confidence interval). The horizontal grey area illustrates the difference between the control and the patient specific conditions. **p < 0.01 and ****p < 0.0001. Mann–Whitney test except for (*H*), Fisher's exact test

The contractile function of control and patient hiPSC‐CM monolayers under physiological conditions (37°C, 5% CO_2_) was assessed using video analysis (Figure 3; Table S2). After 60 days of differentiation, patient hiPSC‐CM demonstrated an increased beating rate (Figure 3B; Table S2). The cellular displacement (amplitude) was higher in the ACM patient cells relative to the control cells (Figure 3C; Table S2). The maximal contraction slope (contraction velocity) was similar in the control and ACM patient cells (Figure 3D; Table S2). The contraction duration (the time from peak contraction to 90% relaxation) was reduced in the ACM hiPSC‐CM monolayers. After correction with Bazett's formula, to account for the difference in beating rates, contraction duration was still reduced in ACM patient cells (Figure 3E; Table S2). The median area under the curve or the median time spent in the resting position did not differ between conditions (Figures 3F and 3G; Table S2). Videos recorded from ACM patient monolayers displayed more aberrant events than the control (35% vs. 25% of the cases, respectively) (Figure 3H; Table S2).

We quantified aberrant events such as changes in the contraction rhythm, and ‘early or delayed after‐contraction’‐like events. Each video was split and recorded as multiple areas beating simultaneously. The percentage of the total area experiencing aberrant events was higher in patient‐derived monolayers (Figure 3I; Table S2). Similarly, the time fraction of each area spent in asynchrony was also increased for ACM patient cells (Figure 3J; Table S2).

### 3.5 DSC2 patient hiPSC‐CM demonstrate altered electrical activity

**FIGURE 4:**
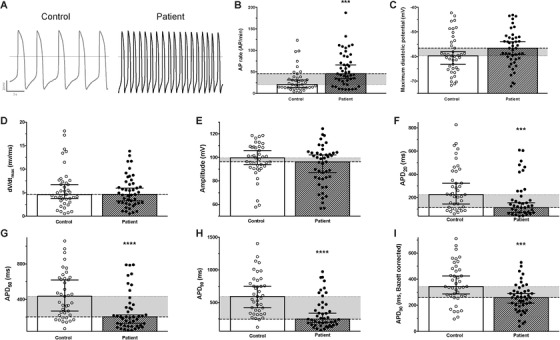
Spontaneous electrical activity of control and patient‐specific hiPSC‐CM. (A) Raw traces illustrating the recording of spontaneous electrical activity (action potentials, AP) of control (left) and patient‐specific (right) hiPSC‐CM. (B**–**I) AP parameters were evaluated for both control (*N* = 41) and ACM patient‐specific (*N* = 45) hiPSC‐CM: (B) AP rate, (C) maximum diastolic potential, (D) maximum depolarization slope, (E) AP Amplitude, (F) AP duration at 20% of repolarization (APD_20_,), (G) APD_50_, (H) APD_90_ and (I) the APD_90_ corrected using Bazett's formula. Histograms represent the median (95% confidence interval). The horizontal grey area illustrates the difference between the control and the patient‐specific conditions. ***p < 0.001 and ****p < 0.0001 (Mann–Whitney test)

We used the patch clamp technique to measure spontaneous APs from control (*N* = 41) and ACM patient‐derived (*N* = 45) single hiPSC‐CM after 60 days of differentiation. We analysed the spontaneous AP rate, the maximum upstroke velocity (*dV*/*dt*_max_), the maximum diastolic potential and the amplitude and the AP duration (APD_20/50/90_) with or without Bazett's formula correction. Results are displayed in Figure 4 and Table S3. APs were also evoked at a fixed pacing rate of 1 Hz, which confirmed these results (Figures S4; Table S4).

The AP maximal upstroke velocity was not affected but the patient‐derived hiPSC‐CM demonstrated a reduced *I*_Na_ density (Figures S5A–C and S6; Table S5). The depolarizing Ca^2+^ channel current, here carried by Ba^2+^ ions to minimise Ca^2+^‐dependent effects, was reduced relative to the control (Figures S5D–F and 6; Table S5). The repolarizing *I*_K_ current was increased, likely accounting for the reduced APD in patient hiPSC‐CM (Figure S5G–I; Table S5).

### 3.6 DSC2 patient hiPSC‐CM demonstrate altered calcium handling

**FIGURE 5:**
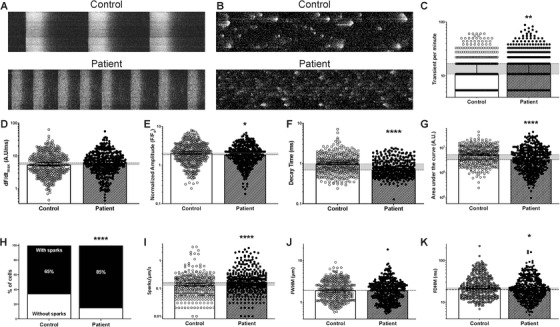
Spontaneous calcium dynamics of control and patient‐specific hiPSC‐CM. (A) Typical line‐scan confocal images (5000 lines, 1.24 ms/line, 1 × 512 pixels) of calcium transients in Fluo‐4 non‐ratiometric fluorescent probe‐loaded control (top) and patient‐specific (bottom) hiPSC‐CM. (B) The global transient activity was removed from the original image to keep only diastolic Ca^2+^ activity (sparks). (C–K). Both the Ca^2+^ transient activity (control *N* = 325 cells; patient *N* = 357 cells) and the sparks (control *N* = 362 cells; patient *N* = 373 cells) were studied: transient per minutes (C), maximum rising fluorescence slope (D), normalised amplitude (E), transient decay duration (F), area under the curve (G), the percentage of cells with Ca^2+^ sparks (control: 65% (362 out of 553); patient specific: 85% (373 out of 441)) (H), in each cell with sparks, their frequency (I), sparks full width at half maximum (J) and sparks full duration half maximum (K). Histograms represent the median (95% confidence interval). The horizontal grey area illustrates the difference between the control and the patient specific conditions. *p < 0.05 and ****p < 0.0001 (Mann–Whitney test, except for (H) where Fisher's exact test was used)

The Ca^2+^ handling properties of spontaneously beating hiPSC‐CM from control (*N* = 362) and patient‐derived (*N* = 373) cells were assessed after 60 days of differentiation using the Fluo‐4 non‐ratiometric Ca^2+^‐sensitive dye. Consistent with both the contractile and the electrical phenotypes, the patient‐derived cells demonstrated an increased frequency of spontaneous Ca^2+^ transients (Figure 5; Table S6). Although the transient rising phase speed (*dF*/*dt*_max_) was not different in patient hiPSC‐CM, the normalised amplitude, the transient decay duration and the area under the curve were reduced relative to the control cell values (Figure 5; Table S6).

Ca^2+^ sparks, as an indicator of ryanodine receptor 2 functional activity, were also assessed. Patient cells demonstrated an increased spontaneous frequency of aberrant Ca^2+^ events (Figure 5; Table S6), and these sparks were also shorter, as illustrated by the reduced full‐duration half maximum (Figure 5; Table S6).

### 3.7 Electrical and contractile disturbances and AADs

The sodium channel blocker flecainide (1 μM) was used to study the Ca^2+^ handling properties in patient hiPSC‐CM differentiated for 60 days (*N* = 331; Figure S7). Flecainide (1 μM) normalised the frequency of spontaneous Ca^2+^ transients to control values, with the normalised amplitude (*F*/*F*_0_) and the area under the curve qualitatively representing the amount of Ca^2+^ mobilised during a Ca^2+^ transient (Figures S7A, S7C and S7E; Table S6). A lower percentage of hiPSC‐CM demonstrated Ca^2+^ sparks in the presence of flecainide (Figure S7F; Table S6), whereas no specific benefits have been observed on their properties (frequency, full‐width half maximum, full‐duration half maximum; Figure S7F and Table S6).

We assessed the effect of 20 μM sotalol on ACM patient‐derived hiPSC‐CM (*N* = 75; Figure S8). Sotalol treatment of ACM patient‐derived hiPSC‐CM partly normalised the AP rate (Figure S8A; Table S3). The maximum diastolic potential, *dV*/*dt*_max_ and AP amplitude values were similar to those of the untreated cells (Figures S8B and S8C; Table S3). Sotalol treatment corrected the APD_20_, APD_50_ and APD_90_ (Figure S8D–F; Table S3). Bazett's correction for the AP rate difference confirmed the APD_90_ normalization (Figure S8G; Table S3). Sotalol also normalised the percentage of recorded cells (28%) demonstrating abnormal electrical events (Figure S8H; Table S3). To confirm the involvement of K^+^ channel blockade, we used the specific E‐4031 (1 μM) blocker confirming an AP lengthening due to K^+^ channel blockade (Figure S9; Table S4). Sotalol normalised the spontaneous beating rate in hiPSC‐CM (Figure S10D; Table S2). This normalization was accompanied by a clear reduction in the number of abnormal contractile events (Figure S10G; Table S2). Similarly, the percentage of the total area experiencing aberrant events and the time fraction of each area in asynchrony were normalised by sotalol (Figures S10H and S10I; Table S2). In similar experiments, propranolol did not reproduce most of the effects observed with sotalol (Figure S10K; Table S2).

This approach was reproduced with four AADs: flecainide, propranolol, sotalol and verapamil (Figure S11). A stepwise, generalised linear model was used to calculate a novel parameter we named ‘differentiating factor’ for each analysed video (Figure S11). This parameter reflects the linear combination of determinant contractile properties ensuring the best discrimination between the control and the patient‐specific condition. Once the linear combination has been identified, it is applied to the patient plus treatment condition to evaluate the treatment benefit (Figure S11B).

### 3.8 ACM as a repolarization disorder disease

The 15‐year patient follow‐up allowed us to evaluate the QT and QRS intervals on surface ECGs. The QRS duration was stable for 5 years at 120 ms and increased to 150 ms during follow‐up (Figure S12). Although QRS lengthening is expected to reflect directly on the QTc duration, the patient QTc decreased strikingly, revealing JT‐segment shortening with disease progression (Figure S12).

**FIGURE 6:**
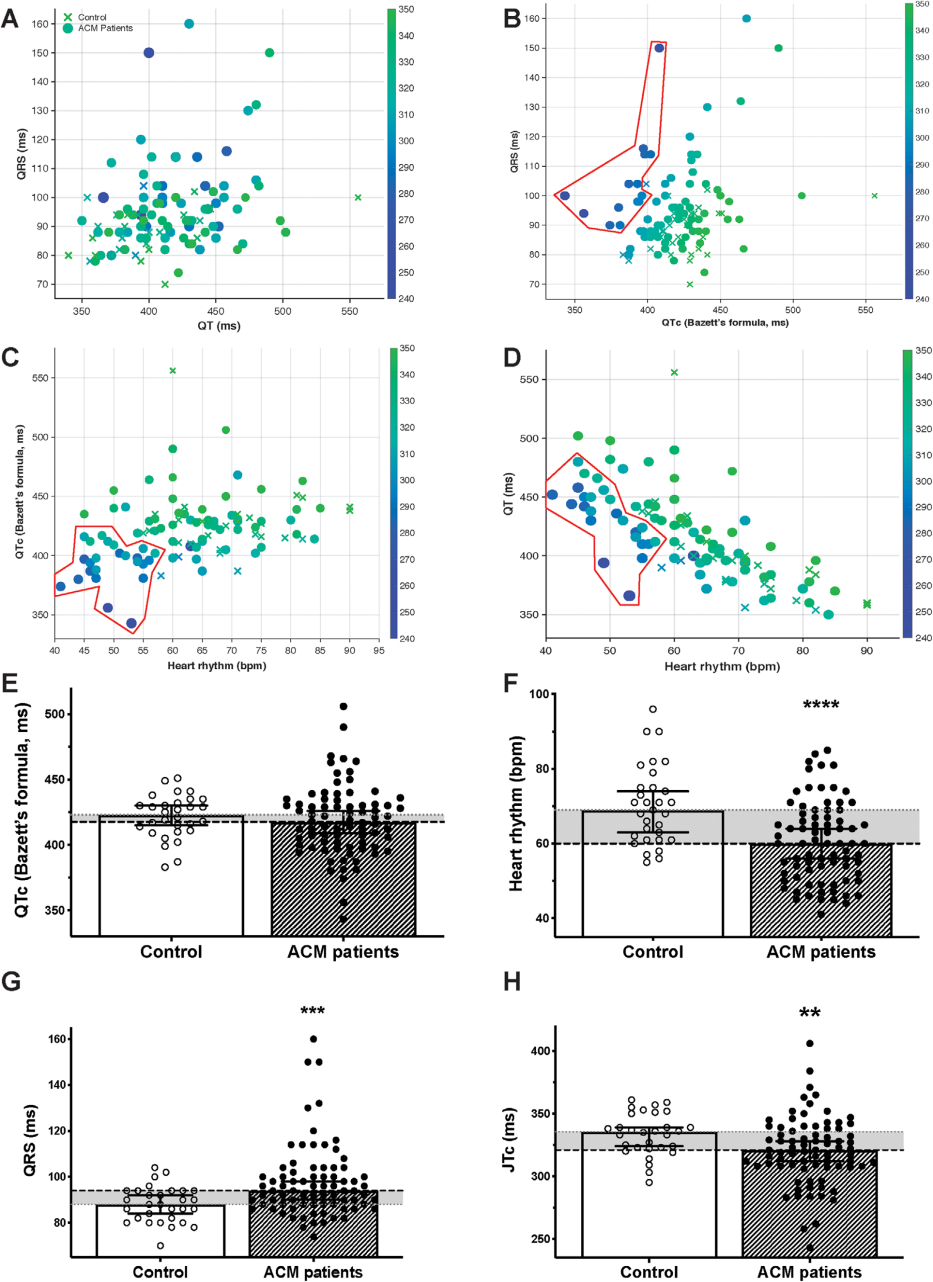
ECG parameters of an ACM cohort. ECG parameters and interdependence in a cohort of control (*N* = 30) versus ACM (*N* = 79) individuals, regardless of their age, genetic variation or treatments. (A–D) Control individuals are represented as crosses and ACM patients as circles. The symbols size (bigger symbols if JTc is longer) and colour both rely on the JTc duration (ms, colour scale indicated on the right of each plot). (A) The QRS duration is plotted as a function of the QT duration. (B) The QRS duration is plotted as a function of the QTc duration (Bazett's formula corrected). (C) The QTc duration (Bazett's formula corrected) is plotted as a function of the heart rate. (D) The QT duration is plotted as a function of the heart rate. (E–H) Recapitulative histograms depicting the control and ACM patient's ECG parameters: the QTc duration (Bazett's formula corrected) (E), the heart rhythm (F), the QRS duration (G) and the JTc duration (H). Histograms represent the median (95% confidence interval). The horizontal grey area illustrates the difference between the control and the patient parameters. *p < 0.05, **p < 0.01, and ****p < 0.0001 (Mann–Whitney test)

The ECG parameters for 79 ACM patients and 30 control individuals were studied (Figure 6; Table S7). For both healthy individuals and ACM patients, the QRS duration did not depend on the QT duration (Figure 6A). Plotting the corrected QT duration against the QRS duration revealed an ACM patient sub‐population with short JT durations, and these were mostly patients with a reduced QTc (Figure 6B). However, the unspecific pattern regarding the QRS duration was noticeable in this sub‐population (Figure 6B). Similarly, the evaluation of QT/QTc dependency on heart rate revealed that the patients with short JT duration were all characterised by low heart rates. This suggests abnormal QT dynamicity, specifically at reduced heart rate (Figures 6C and 6D). The QTc interval of ACM patients did not differ from controls (controls: 69 (± 63/74) bpm; patients: 60 (± 56/74) bpm) (Figures 6E and 6F). The QTc was similar but the QRS duration was increased in ACM patients (controls: 88 (± 84/92) ms; patients: 94 (± 90/98) ms), with a shorter JTc duration (controls: 335.5 (± 324/339) ms; patients: 321.5 (± 314/328) ms) (Figures 6G and 6H).

The cycle‐length effect was evaluated on the hiPSC‐CM model through both elicited and spontaneous AP (Figure S13). Although the APD_90_ was already shorter in ACM hiPSC‐CM, these patient‐specific hiPSC‐CM revealed significant abnormal AP dynamicity at low stimulation frequencies (Figures S13A and S13B). These results were confirmed by plotting the spontaneous period between APs against the APD_90_ for control and patient‐derived cells (Figure S13C). A shorter APD for ACM patient hiPSC‐CM with a low spontaneous AP firing rate was found (Figure S13C).

## 4 DISCUSSION

In this study, the pathogenicity of the *DSC2* p.R132C substitution was confirmed in the zebrafish model. The use of hiPSC‐CM derived from the *DSC2* patient demonstrated that (1) arrhythmias constitute a direct pathological mechanism and are not a consequence of tissue remodelling during disease progression; (2) both electrical activity and Ca^2+^ handling are impaired, leading to contractile dysfunction; and (3) AP duration was shortened, revealing repolarization issues in many ACM patients’ ECGs. Finally, our data establish a rationale for the use of AADs in ACM patients.

### 4.1 DSC2/R132C as a disease‐causing substitution

Desmosomes are mainly found in organs exposed to frequent mechanical stress such as skin and heart.^30^ Gehmlich et al. identified two *DSC2* missense mutations (encoding R203C and T275M) causing altered proteolytic cleavage.^7^ The DSC2/R132 residue is the first arginine of the protease recognition site. Its replacement by a cysteine might prevent proteolysis, resulting in an inactive desmocollin. Interestingly, data from both the patient's heart and hiPSC‐CM revealed that the DSC2/R132C substitution not only converts desmocollin to a non‐adherent protein but also induces a marked loss of *DSC2* mRNA expression.

The zebrafish larva has been used to model the pathogenicity of *DSC2* variants linked to ACM at the organ level.^9^, ^31^ Knockdown of the native fish dsc2 enabled the study of cardiac behaviour in the setting of control or patient‐specific *DSC2* expression. This model confirmed the specific pathogenicity of the DSC2/R132C substitution.

### 4.2 ACM: A myogenic electrical disease?

Fibro‐fatty replacement, a hallmark of ACM, was thought to be the main pro‐arrhythmogenic mechanism in this disease. Few studies have focused on the electrical features of ACM cardiomyocytes.^3^, ^32^, ^33^ We found a shortened AP, explained by both an increased density of the voltage‐gated K^+^ current and a decreased density of the voltage‐gated Ca^2+^ current. The increase in K^+^ current density was consistent with recent findings using hiPSC‐CM derived from a *DSG2* patient, yet the AP duration was unmodified in that study.^34^

Using video analysis, we found an increased spontaneous beating rate, which was confirmed with the finding of a higher rate of spontaneous AP firing. This was somewhat surprising, because the observed reduced Na^+^ current density, in agreement with previous reports, would favour rather a lower cellular excitability.^34^, ^35^ The spontaneous increase in cellular excitability could rely on ß‐adrenergic pathway basal overactivity. This is suggested by the decrease in beating rate caused by both sotalol and propranolol. Although propranolol reduced the spontaneous hiPSC‐CM beating rate, it failed to correct contraction duration and contractile aberrant events. This would thus indicate that as sotalol, propranolol is efficient to correct the ß‐adrenergic over‐activity in the patient‐specific model, but that this overactivity is not mainly responsible for aberrant contractile events. Such an increase in cellular excitability may also result from altered Ca^2+^‐handling dynamics. The ryanodine receptor (RyR2) is a Ca^2+^ channel located at the surface of the sarcoplasmic reticulum. It is responsible for the massive intracellular Ca^2+^ release that triggers the Ca^2+^ transient supporting the contraction. Although they are very rare, RyR2 genetic variants have been identified in ACM patients, consistent with unbalanced Ca^2+^ handling in ACM.^36^, ^37^, ^38^ We determined that patient‐derived hiPSC‐CM exhibited both reduced global Ca^2+^ mobilization and spontaneous aberrant microscopic diastolic Ca^2+^ events likely to be involved in ACM arrhythmogenesis. This is in agreement with recent experimental data from a novel murine model with cardiac‐specific, tamoxifen‐triggered PKP2 deficiency: PKP2 deficiency increased RyR2‐mediated Ca^2+^ release from the sarcoplasmic reticulum, leading to catecholamine‐induced arrhythmias.^39^

### 4.3 AAD effects: From ion channels to ECG

Studies on AAD prescription for ACM patients are lacking. In our model, hiPSC‐CM mechanical properties recapitulated their electrical features and Ca^2+^ handling. The stepwise, generalised linear model identified the combination of parameters that best differentiated control from patient cell monolayer properties. Among the four tested drugs, sotalol and flecainide were clearly the most effective in normalizing hiPSC‐CM properties. Clinical interest in sotalol is often reported but evidence supporting its use is sparse.^17^, ^40^, ^41^, ^42^, ^43^, ^44^ Although sotalol is known to trigger torsade de pointe, this dramatic adverse effect has not been reported in ACM patients, even when sotalol has been combined with a class Ia molecule (flecainide). In the *DSC2* hiPSC‐CM cells, both electrical activity and mechanical function were improved by sotalol.

Flecainide was proposed as a therapeutic to prevent diastolic Ca^2+^ waves and arrhythmias in catecholaminergic polymorphic VT.^45^, ^46^, ^47^, ^48^ Here, flecainide normalised the frequency of spontaneous Ca^2+^ transients and the occurrence of Ca^2+^ sparks in *DSC2* hiPSC‐CM cells, providing a rationale for its therapeutic use in ACM. Our results are in line with recently published findings of dysregulation of intracellular Ca^2+^ homeostasis and effective therapy with flecainide in a PKP2‐deficient mouse model.^39^ These studies have motivated a multicentre, pilot clinical trial that the NIH recently funded (https://projectreporter.nih.gov/project_info_description.cfm?aid=9587014&icde=45130245&ddparam=&ddvalue=&ddsub=&cr=4&csb=default&cs=ASC&pball=).

### 4.4 A repolarization disorder

It has been suggested that symptomatic ventricular arrhythmia begins during the electrical phase of ACM. A recent study reported two patients with apparently normal hearts presenting ventricular fibrillation in early‐stage ACM.^49^ The *PKP2c*KO murine model developed by Cerrone et al. supports a myogenic origin of VA in ACM patients.^39^ The shortened APD we observed led us to identify a previously unknown abnormal repolarization dynamicity in some patients with a QT‐interval not lengthening as expected at low heart rate. In most patients with ACM, right‐ventricular parietal block, reduced QRS amplitude, epsilon wave, and T‐wave inversion in V1‐3 are present, but QT‐ or JT‐interval dynamicity has never been looked for. A short QT interval may have been overlooked because of QRS widening and masking this abnormality, mainly at slow heart rates. In addition, ACM being an evolutive disease, short myocardial repolarization times may be obvious only during a brief time frame and may be blurred by drug treatment. The hypothesis of an increased repolarization reserve would also be strengthened by the high efficacy of sotalol in some ACM patients, and high drug dosage (320–480 vs. 160–320 mg/day) has been shown to have a greater antiarrhythmic efficacy.^42^, ^44^ The reverse‐use dependence effect of sotalol may underlie its efficacy and its safety in a setting of an increased repolarization reserve.^50^ In the present case, with right‐dominant ACM, the short coupling interval of the ventricular fibrillation triggered by ventricular premature beats also suggests a shortened refractory period and supports the use of QT‐prolonging drugs in ACM patients.

### 4.5 Study limitations

The starting point of this study was based on a single patient and a single genetic variation located on the *DSC2* gene. Although this may limit our conclusions on this single genetic variation, we also studied a cohort of ACM patients, regardless of their genetic variations. As such, the subpopulation of ACM patients with altered QT dynamicity does not exclusively harbour *DSC2* genetic variations, thus demonstrating a general process not only focused on the defect in the *DSC2* gene. Regarding RNA sequencing data, we also acknowledge a limited statistical power due to difficulties in accessing the valuable biological samples (control and patient heart samples). Although these data are not critical to the overall message, we believe that they can be valuable to other researchers within the field to compare with their own datasets. Recent gene editing technologies could be used to generate isogenic cell models. Using this technology, it is possible to compare two cell lines in which the only theoretical difference is the presence (or absence) of a patient‐specific genetic variation. In our case, the use of these technologies could be used to clearly establish the pathogenicity of a genetic variation found in a patient. In our study, for this, we chose to focus on a more integrative model (i.e., zebrafish), which allowed us to assess the specific effect of genetic variation at the level of organs and organisms.

## 5 CONCLUSION

A multi‐faceted functional study of a novel *DSC2* variant combining clinical, histological, zebrafish and hiPSC data helped to define ACM pathology. Patient‐specific hiPSC‐CM revealed major electrical disturbances with shorter APD supporting abnormal repolarization dynamicity. By normalizing the increased repolarization reserve of ACM myocytes, class 3 AADs are likely to be the first drugs prescribed for *DSC2* patients. Because flecainide normalised Ca^2+^‐related events, it may be used as a second choice or in combination with a class 3 AAD. These findings may encourage the establishment of randomised, controlled trials to evaluate class 1 or pure class 3 AADs in ACM patients.

## CONFLICT OF INTEREST

The authors declare no conflict of interest.

## Supporting information

Supporting information

Supplemental Video 1

Supplemental Video 2

Supplemental Video 3

Supplemental Video 4

Supplemental Video 5
