# Supplementary material for: Deciphering DSC2 arrhythmogenic cardiomyopathy electrical instability: From ion channels to ECG and tailored drug therapy
Source: Clin Transl Med. 2021 Feb 26;11(3):e319. doi: 10.1002/ctm2.319 (PMC7908047; doi:10.1002/ctm2.319)
Supplement: Supplementary file 1 — Supporting information [file CTM2-11-e319-s001.pdf]

Deciphering DSC2 arrhythmogenic cardiomyopathy electrical instability: from ion channels to ECG and tailored drug therapy

Online Data Supplements

The data that support the findings of this study are available from the corresponding author upon reasonable request.

Additional Tables:

Supplementary Table 1: Zebrafish heart study

	Not injected (n=28)	KD ATG (n=30)	KD MO (n=28)	KD MO + Control mRNA (n=24)	KD MO + Patient mRNA (n=23)
**Heart beat (bpm)**	160 (153/167)	152.5 (140/160)	157.5 (152/165)	163 (153/171)	153 (151/162)
**Blood flow (nL/sec)**	0.15 (0.10/0.17)	0.05 (0.04/0.07)	0.06 (0.05/0.08)	0.09 (0.08/0.1)	0.05 (0.03/0.06)
**Stroke Volume (nL/beat)**	0.048 (0.035/0.060)	0.019 (0.013/0.027)	0.023 (0.017/0.030)	0.032 (0.028/0.039)	0.017 (0.013/0.025)
**Atrium Contractility (µM)**	23 (21.8/25)	22.7 (19.5/23.4)	22 (18.7/24.2)	19.5 (17.3/23.4)	18.6 (17.5/21.7)
**Ventricle Contractility (µM)**	21.5 (19.5/23.6)	10.7 (6/12.2)	8.1 (4.5/12.5)	17.5 (14.7/19.5)	6.1 (14.5)

Data are expressed as median (95% interval of confidence).

Supplementary Table 2: 60 days old control and patient contractile characteristics

	Control (n=229)	Patient (n=359)	Patient + 20µM Sotalol (n=129 videos)	Patient + 3µM propranolol (n=105 videos)
**Beat rate (bpm)**	91 (88.2/96.6)	113.3 (101.5/123.3) **	114.3 (51.2/123.8)	64.9 (62.6/66.4) ****/####
**Displacement (µm)**	2.53 (2.28/2.73)	2.81 (2.55/3.03) **	3.56 (3.02/3.86) ****/##	6.97 (5.58/8.02) ****/####
**Contraction velocity (µm/ms)**	25.3 (23.6/27.6)	27.1 (24/31)	26.9 (22.4/31)	62.3 (55.9/76.6) ****/####
**Contraction $Duration_{90}$ (ms)**	252 (240/269)	181 (175/198) ****	231 (213/259) ####	179 (159/218) ****
**Contraction $Duration_{90}$ Bazett corrected (ms)**	314 (296/335)	254 (250/258) ****	311 (300/324) ####	186 (166/203) ****/####
**Area under the curve (A.U.)**	4.9 (4.5/4.3)	5.1 (4.8/5.6)	7.1 (5.6/9.4) ***/##	8.2 (5.8/11.7) ****/####
**Resting duration (ms)**	255 (231/273)	217 (201/238)	197 (162/451)	445 (388/518) ****/####
**Traces with aberrant contractions (%)**	25 (278 out of 1097)	35 (600 out of 1727) ****	23 (141 out of 614) ####	35 (172 out of 497) ***
**Asynchrony rate (%) of each trace**	9.6 (7.8/11.2)	11.8 (10.3/12.9) **	8 (6.2/9.6) #	19.1 (16.9/21.9) ****/####
**Asynch. percent (% of each video with aberrant contractions)**	9 (6/11)	16 (14/17) ****	9 (6/13) ###	13 (10/17)

Data are expressed as median (95% interval of confidence).
Differences were evaluated using a non-parametric Kruskal-Walis test (with Dunn's correction for multiple comparisons). * indicate differences with control condition, # indicate differences with patient condition. Exception: for "traces with aberrant contractions (%)", the Fisher exact test was used to compare the two conditions. */# p<0.05, **/## p<0.01, ***/### p<0.001, ****/#### p<0.0001

Supplementary Table 3: 60 days old control and patient spontaneous electrical activity

	**Control (n=41)**	**Patient (n=45)**	**Patient + 20 µM sotalol (n=75)**
**AP rate (AP/min)**	21 (14/31)	46 (34/66) *******	34 (27/41) *****
**Max. diastolic potential (mV)**	-59.7 (-63.2/-58)	-56.6 (-59.2/-54)	-62.1 (-64.1/-59.4) ##
**dV/dt_{max} (mV/ms)**	4.6 (3.8/6.7)	4.6 (3.3/6)	5 (3.4/6.2)
**Amplitude (mV)**	99.6 (94.1/105.8)	96.3 (87.1/102.2)	98.9 (94.3/103.8)
**APD_{20} (ms)**	225.2 (145.6/323.6)	113.2 (72.4/154.6) *******	153 (127.4/183.7)
**APD_{50} (ms)**	435.4 (269.4/620.6)	203.6 (131.6/226.2) ********	282.8 (229.8/316.6) #
**APD_{90} (ms)**	593.3 (422.7/751.4)	246.2 (196.9/338.6) ********	413.9 (375.4/465.6) ###
**APD_{90} Bazett corrected (ms)**	343.2 (287.6/424.7)	259 (216.5/289.9) *******	309.7 (280/335.3) ##
**Traces with aberrant events (%)**	22 (9 out of 41)	50 (24 out of 48) ******	28 (21 out of 75) #

Data are expressed as median (95% interval of confidence).
Differences were evaluated using a non-parametric Kruskal-Walis test (with Dunn's correction for multiple comparisons) and ***** indicate differences with control condition, **#** indicate differences with patient condition. Exception: for "Traces with aberrant events (%)", the Fisher exact test was used to compare the two conditions. */# p<0.05, **/## p<0.01, ***/### p<0.001, ****/#### p<0.0001

Supplementary Table 4: 60 days old control and patient evoked electrical activity (1 Hz)

	**Control (n=64)**	**Patient (n=60)**	**Patient + 1 µM E-4031 (n=48)**
**dV/dt_{max} (mV/ms)**	81.2 (63.3/99.6)	80.6 (71.8/90.2)	73.3 (54.3/88)
**Amplitude (mV)**	122.3 (116.1/125.2)	119.3 (114.9/121.9)	117.2 (113.7/120.9)
**APD_{20} (ms)**	110.1 (86.6/140.8)	50.9 (16.7/96.4) ******	172.1 (90.8/199.9) ####
**APD_{50} (ms)**	203.7 (147.7/255.6)	107.7 (48.2/182.1) ******	262.5 (205.9/353.1) *** /** ####
**APD_{90} (ms)**	351.3 (256.9/435.8)	183.4 (123.7/281.7) *******	518 (449.8/593.3) ***** /** ####

Data are expressed as median (95% interval of confidence).
Differences were evaluated using a non-parametric Kruskal-Walis test (with Dunn's correction for multiple comparisons). ***** indicate differences with control condition, **#** indicate differences with patient condition. */# p<0.05, **/## p<0.01, ***/### p<0.001, ****/#### p<0.0001

Supplementary Table 5: 60 days old control and patient ion channels parameters

	**Control**	**Patient**
**Na_v Channels**	(n=20)	(n=31)
Reversal potential (mV)	26.8 (22.3/28.2)	23.6 (21.4/26.6)
Normalized maximal conductance	10.1 (6.5/15.1)	5.4 (3.5/9.3) *****
Normalized maximal current (pA/pF)	-455.1 (-640.9/-332.5)	-231.2 (-267.9/-146.8) ******
$V_{1/2}$ activation (mV)	-37.4 (-42.8/-30.9)	-31.6 (-37/-28.3)
Slope (k) activation (mV)	-3.7 (-9.1/-2.9)	-7.3 (-9.5/-4.8)
$V_{1/2}$ inactivation (mV)	-71 (-74.6/-65.5)	-72.1 (-73.9/-70.4)
Slope (k) inactivation (mV)	5.4 (5.1/5.7)	4.8 (4.7/5.1)
**Ca_v Channels**	(n=256)	(n=127)
Reversal potential (mV)	41.9 (41.3/42.5)	43 (42/43.9)
Normalized maximal conductance	0.60 (0.55/0.64)	0.46 (0.40/0.52) ********
Normalized maximal current (pA/pF)	-21.4 (-24/-19.4)	-17 (-19.3/-12.7) ********
$V_{1/2}$ activation (mV)	-12.2 (-12.7/-11.7)	-10.8 (-11.6/-10.1) *******
Slope (k) activation (mV)	-5.4 (-5.2/-5.6)	-5.6 (-5.4/-5.9)
**K_v Channels**	(n=19)	(n=23)
Normalized maximal current (pA/pF)	1.8 (0.9/4.2)	5.3 (3.4/11.3) *******

Data are expressed as median (95% interval of confidence).
Differences were evaluated using a non-parametric Mann Withney test and starts indicate differences with control condition. * p<0.05, ** p<0.01, *** p<0.001, **** p<0.0001

Supplementary Table 6: 60 days old control and patient spontaneous Ca^{2+} activity

	**Control (n=403 cells)**	**Patient (n=357 cells)**	**Patient + 1µM flecainide (n=306 cells)**
**Transient rate (Transient/min)**	10.8 (10.8/16.8)	16.8 (10.8/16.8) ******	10.8 (10.8/16.8)
**dF/dt_{max} (mV/ms)**	5.4 (5.0/6.4)	6.1 (5.5/6.9)	6.7 (6.1/7.3) ******
**Normalized Amplitude ($\Delta F/F_0$)**	2.1 (1.9/2.3)	1.9 (1.7/2) *****	2.1 (2.0/2.3) #
**Decay duration (ms)**	1 (0.9/1.1)	0.7 (0.7/0.7) ********	0.8 (0.8/0.9) ********
**Area under the curve (A.U)**	$5.2.10^6$ ($4.9.10^6$/$5.8.10^6$)	$3.3.10^6$ ($2.8.10^6$/4.10^6) ********	$4.8.10^6$ ($4.3.10^6$/$5.4.10^6$) ####
**Cells with sparks (%)**	65 (362 out of 553)	85 (373 out of 441) ********	79 (321 out of 407) ******/#**
**Sparks frequency (sparks/µm/s)**	0.13 (0.12/0.15)	0.16 (0.14/0.18) ********	0.15 (0.14/0.16) *****
**Sparks FWHM (µm)**	1.9 (1.7/2.1)	1.9 (1.8/2.1)	2.4 (2.3/2.6) ******/####**
**Sparks FDHM (ms)**	22.2 (20.9/24.1)	20.2 (19.1/21.7) *****	26.3 (24.4/29.4) ****/####**

Data are expressed as median (95% interval of confidence).
Differences were evaluated using a non-parametric Kruskal-Walis test (with Dunn's correction for multiple comparisons) and ***** indicate differences with control condition, # indicate differences with patient condition. Exception: for "Cells with sparks (%)", the Fisher exact test was used to compare the two conditions. */# p<0.05, **/## p<0.01, ***/### p<0.001, ****/#### p<0.0001

Supplementary Table 7: ACM patients ECG parameters

	Controls (n=31)	ACM Patients (n=78)
**Heart Rhythm (bpm)**	69 (63/74)	60 (56/64) ******** (p<0.0001)
**QRS (ms)**	88 (84/92)	94 (90/98) ******* (p=0.0009)
**QT (ms)**	393 (380/398)	418 (406/430) ******** (p<0.0001)
**QTc (Bazett's formula, ms)**	423 (415/430)	418 (409/426) (p=0.3141)
**JTc (ms)**	336 (324/339)	321 (312/328) ****** (p=0.0076)

Data are expressed as median (95% interval of confidence).
Differences were evaluated using a non-parametric Mann Withney test and starts indicate differences with control condition.

Additional Figure:

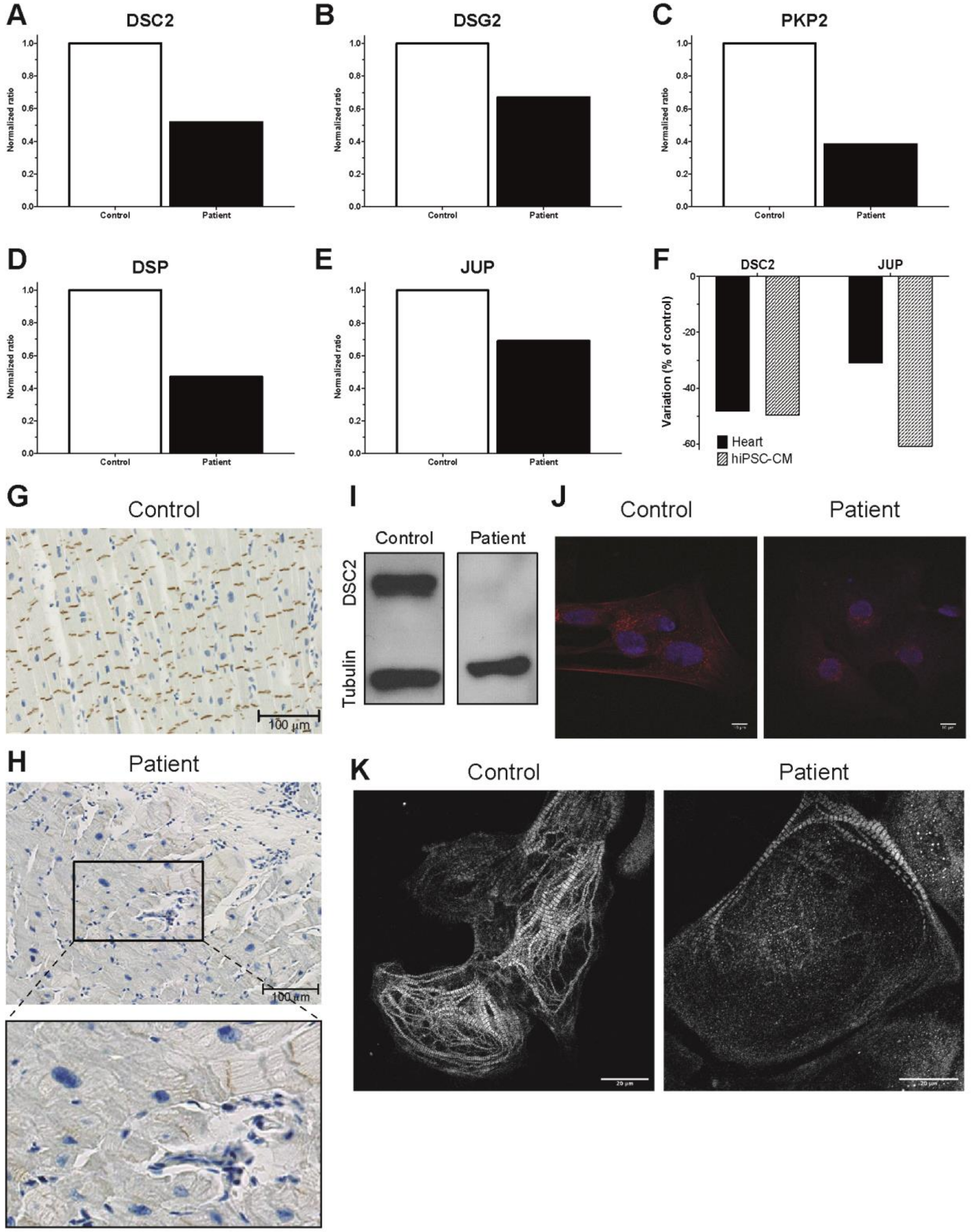

Supplementary Figure 1: Molecular and cellular impairment of patient's heart and hiPSC-CM

A-E. Levels of mRNA coding for desmosomal protein between a control right ventricle (RV) *vs.* the ACM patient explanted RV: DSC2 (A.), DSG2 (B.), DSP (C.), PKP2 (D.), et JUP (E.). Levels are normalized to the expression level of the control RV. **F.** Variations (expressed as percentage of control) of expression levels of DSC2 and JUP mRNA between the control *vs.* patient RV and between the controls (n=9) *vs.* patient specific (n=6) hiPSC-CM. **G-H.** Control (G) and patient (H) RV microsection illustrating a strong cellular disorganization associated with a loss of JUP expression at intercalated disks in the patient RV. **I.** DSC2 protein expression level from control and patient's explanted heart. Tubulin is used as protein level marker. **J.** DSC2 immunostaining of control and patient specific hiPSC-CM showing a strong loss of DSC2 labelling in the patient cells, associated with a perinuclear location of the DSC2 protein. **K.** Myosin light chain 2v immunostaining of control and patient specific hiPSC-CM illustrating the sarcomeric organization. Such organization is partially lost in patient cells.

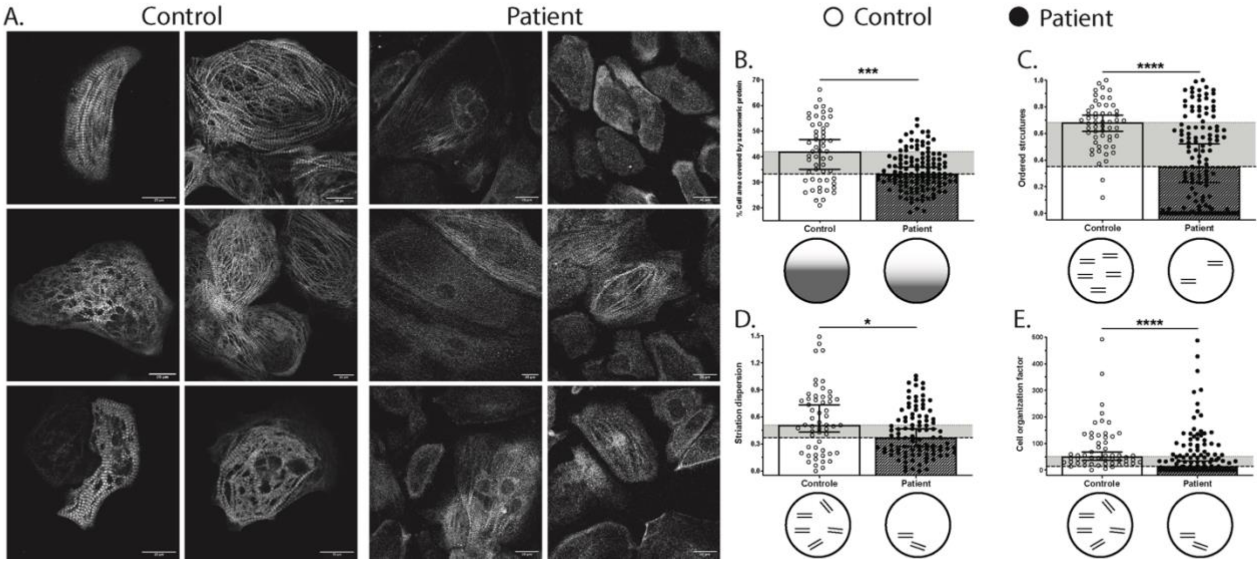

Supplementary Figure 2: hiPSC-CM sarcomeric organization.

A. Pictures of mlc2v immunolabeled control (left) and patient specific (right) hiPSC-CM. **B-E.** Custom made Matlab routine analysis was specifically made to study the hiPSC-CM sarcomeric organization of control (n=55) and patient specific (n=129) hiPSC-CM. All parameters are schematically illustrated under each graphs. The mlc2v protein coverage (**B**) and its organization as doublets (**C**) are studied. Concerning surfaces covered by sarcomeres, their spatial orientation is also studied (**D**). **E.** The cell organization factor is defined by a large cell area of organized sarcomeric proteins, with the same orientation, as illustrated by the proposed "cell organization factor": ((organized areas^3)*(1/Striation dispersion))*100. Histograms represent the median (the 95% confidence interval). The horizontal grey area illustrates the difference between the control and the patient specific conditions. *$p<0.05$, ***$p<0.001$, ****$p<0.0001$ (Mann-Whitney test).

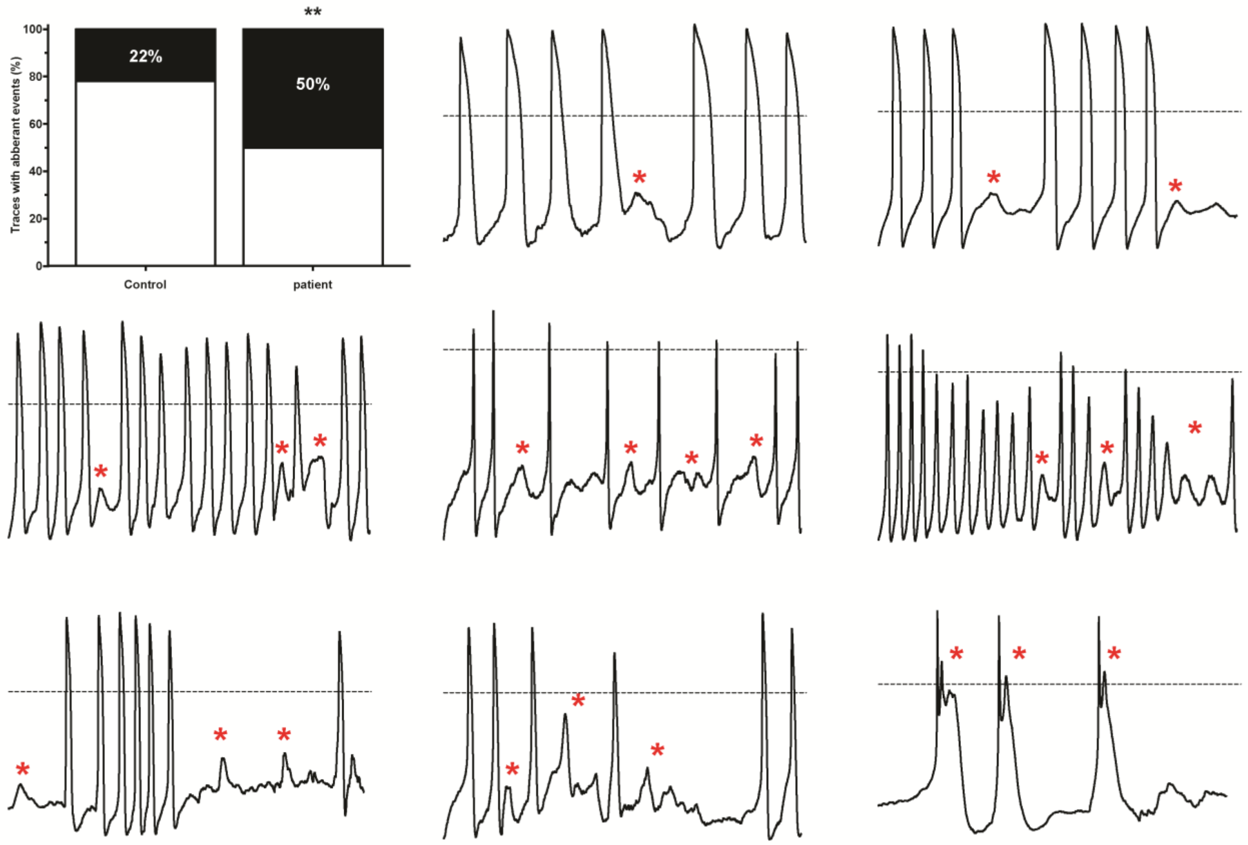

Supplementary Figure 3: Control and patient specific hiPSC-CM spontaneous aberrant electrical activity

ACM patient specific hiPSC-CM spontaneously demonstrated increased aberrant electrical activity (top left) (control: 22% (9 out of 41); patient specific: 50% (24 out of 48)), as illustrated by the raw traces. Red stars highlight aberrant events. **p<0.01 (Fisher's exact test).

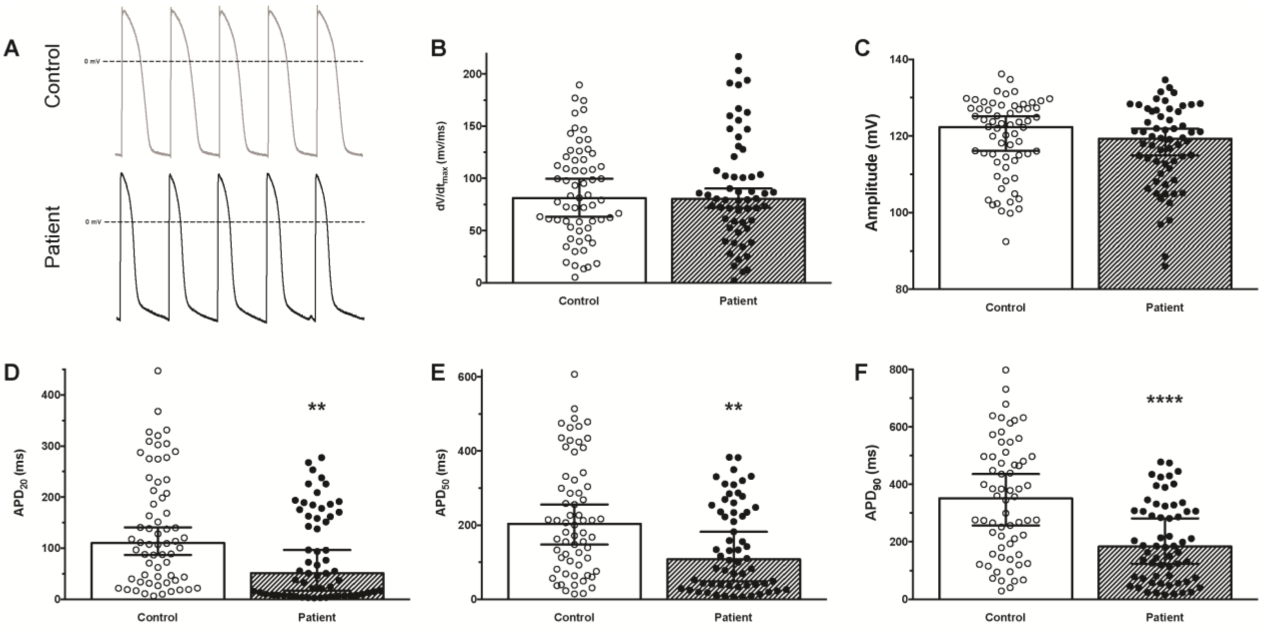

Supplementary Figure 4: Evaluation of control and patient specific hiPSC-CM paced electrical activity

A. Raw traces illustrating the recording of evoked electrical activity at 1Hz (action potentials, AP) of control (top) and patient specific (bottom) hiPSC-CM. **B-F.** AP parameter were evaluated for both control (n=64) and ACM patient specific (n=60) hiPSC-CM: maximum depolarization slope (**B**), AP Amplitude (**C**), AP duration at 20% of repolarization (APD_{20}, **D**), APD_{50} (**E**), APD_{90} (**F**). Histograms represent the median (95% confidence interval). **$p<0.01$, ****$p<0.0001$ (Mann-Whitney test).

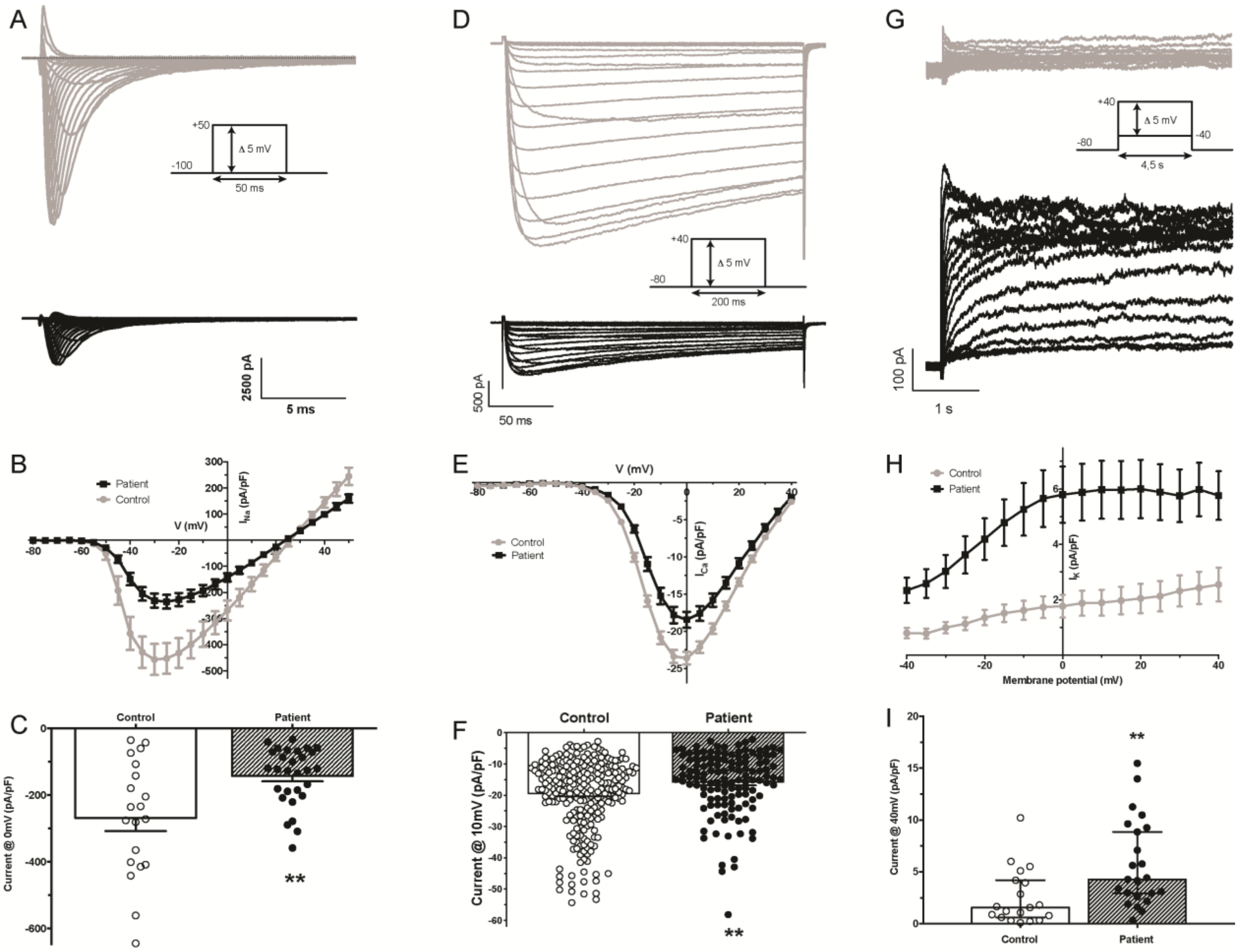

Supplementary Figure 5: Evaluation of control and patient specific hiPSC-CM main voltage gated ion channels

A-C. Voltage gated sodium channels are studied in control (n=20) and ACM patient specific (n=31) hiPSC-CM. **A**. Representative whole-cell current traces of control (top) and ACM patient specific (bottom) hiPSC-CM. Currents were elicited using a voltage-clamp protocol where depolarizing pulses were applied for 50 ms from -100 to +50 mV in 5 mV increments (see protocol in inset). **B**. Current density-voltage (I-V) relationship of the control (grey) and ACM patient specific (black) hiPSC-CM. **C**. The current density is compared at 0 mV where the channel activation is completed. **D-F.** Voltage gated calcium channels are studied as Ba^{2+} currents in control (n=256) and ACM patient specific (n=127) hiPSC-CM. **D**. Representative whole-cell current traces of control (top) and ACM patient specific (bottom) hiPSC-CM. Currents were elicited using a voltage-clamp protocol where depolarizing pulses were applied for 200 ms from -80 to +40 mV in 5 mV increments (see protocol in inset). **E**. Current density-voltage (I-V) relationship of the control (grey) and ACM patient specific (black) hiPSC-CM. **F**. The current density is compared at +10 mV where the channel activation is completed. **G-I.** Voltage gated potassium channels are studied in control (n=19) and ACM patient specific (n=23) hiPSC-CM. **G**. Representative whole-cell current traces of control (top) and ACM patient specific (bottom) hiPSC-CM. Currents were elicited using a voltage-clamp protocol where depolarizing pulses were applied for 4.5 s from -40 to +40 mV in 5 mV increments (see protocol in inset). **H**. Current density-voltage (I-V) relationship of the control (grey) and ACM patient specific (black) hiPSC-CM. **I**. The current density is

compared at +40 mV where the channel activation is completed. Histograms represent the mean ± SEM. **p<0.01 (student t-test).

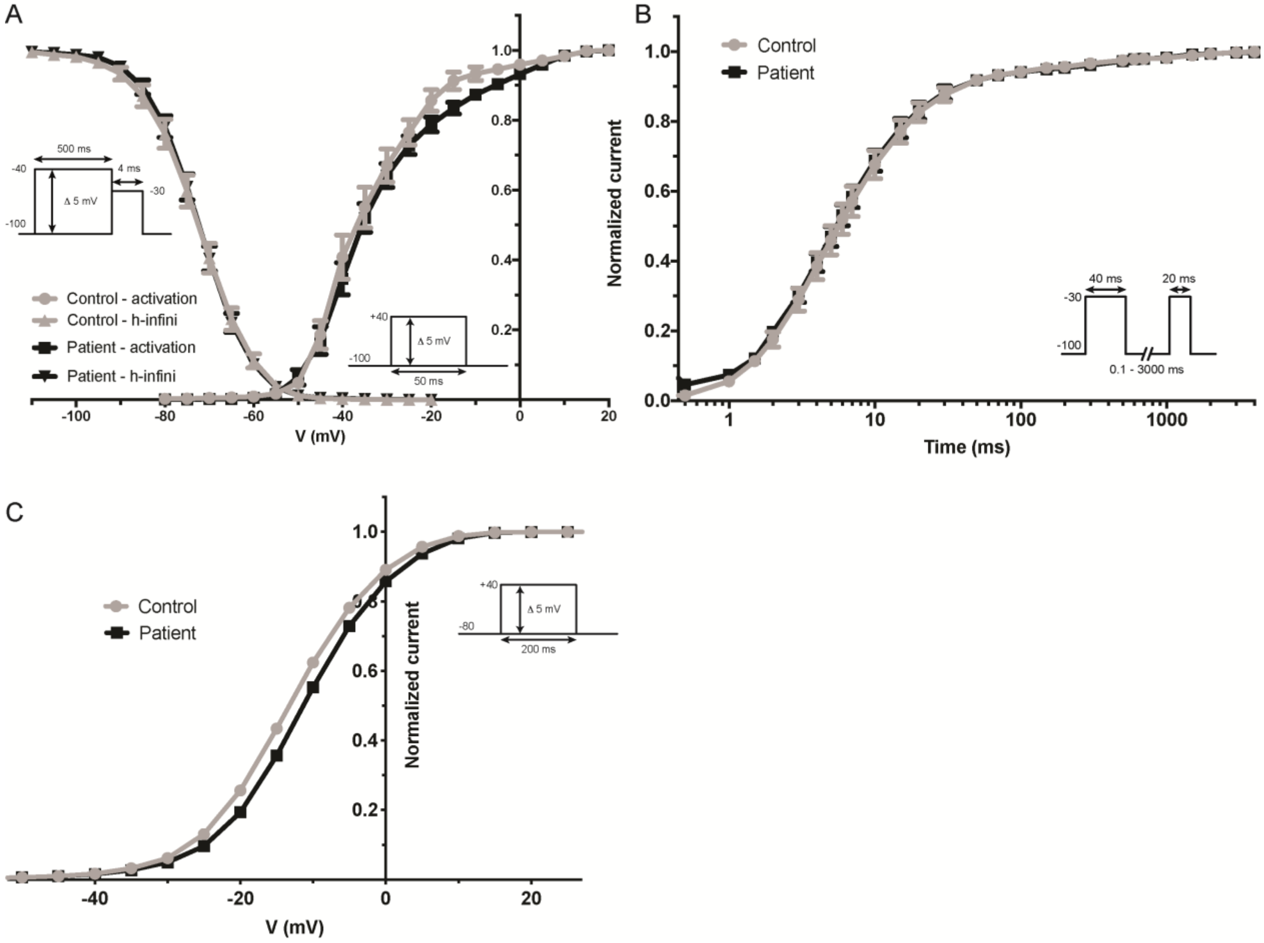

Supplementary Figure 6: Evaluation of control and patient specific hiPSC-CM Na_v and Ca_v biophysical parameters

A. Voltage-dependence of steady-state activation and inactivation of Na_v channels in control (grey) and ACM patient specific (black) hiPSC-CM. Activation curves were generated using a standard Boltzmann distribution [G(V)/Gmax = 1/(1 + exp(-(V - V1/2)/k))]. Inactivation currents were obtained by applying conditioning pre–pulses to membrane potentials ranging from a holding potential of -100 to -40 mV for 500 ms in 5 mV increments and were then measured using a 4-ms pulse to -30 mV at each step (see protocol in inset). The recorded inactivation values were fitted to a standard Boltzmann equation [I(V)/Imax = 1/(1 + exp((V - V1/2)/k)) + C]. **B.** Recovery from fast inactivation was obtained using a two–pulse protocol at +30mV to obtain maximal activation (see protocol in inset). **C.** Voltage-dependence of steady-state activation of Ca_v channels in control (grey) and ACM patient specific (black) hiPSC-CM. Activation curves were generated using a standard Boltzmann distribution [G(V)/Gmax = 1/(1 + exp(-(V - V1/2)/k))].

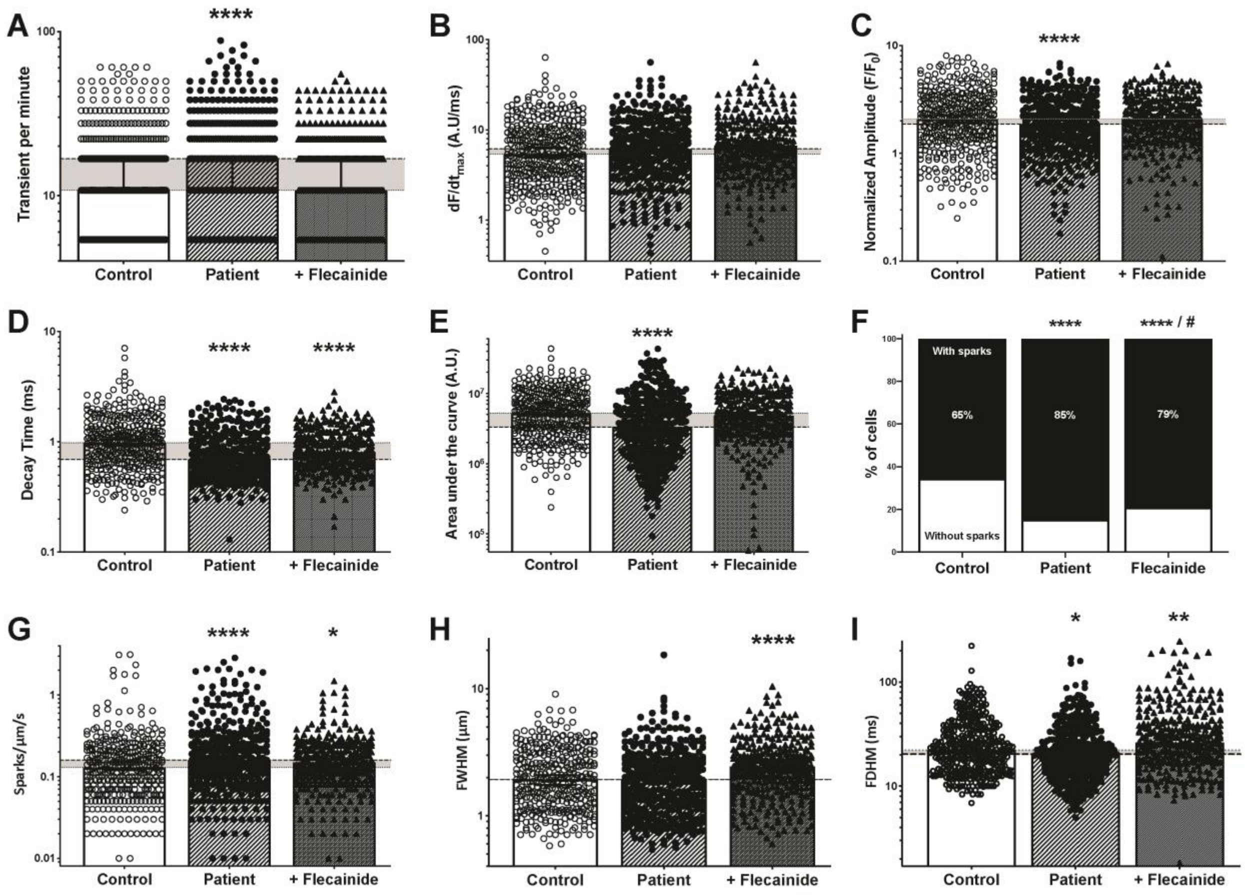

Supplementary Figure 7: Evaluation of the flecainide treatment on spontaneous calcium dynamics

A-I. Both the Ca^{2+} transient activity (control n= 325 cells; patient n=357 cells; patient+1µM Flecainide n=261 cells) and the sparks (control n= 362 cells; patient n=373 cells; patient+1µM Flecainide n=285 cells) were studied: transient per minutes (**A**), maximum rising fluorescence slope (**B**), normalized amplitude (**C**), transient decay duration (**D**), area under the curve (**E**), the percentage of cells demonstrating Ca^{2+} sparks (control: 65% (362 out of 553); patient specific: 85% (373 out of 441); patient specific + flecainide: 79% (321 out of 407)) (**F**), in each cell with sparks, their frequency (**G**), sparks full width at half maximum (**H**), sparks full duration half maximum (**I**). Histograms represent the median (95% confidence interval). The horizontal grey area illustrates the difference between the control and the patient specific conditions. *p<0.05, **p<0.01, ***p<0.001, ****p<0.0001 (Kruskal-Wallis test, except for (F): Fisher's exact test).

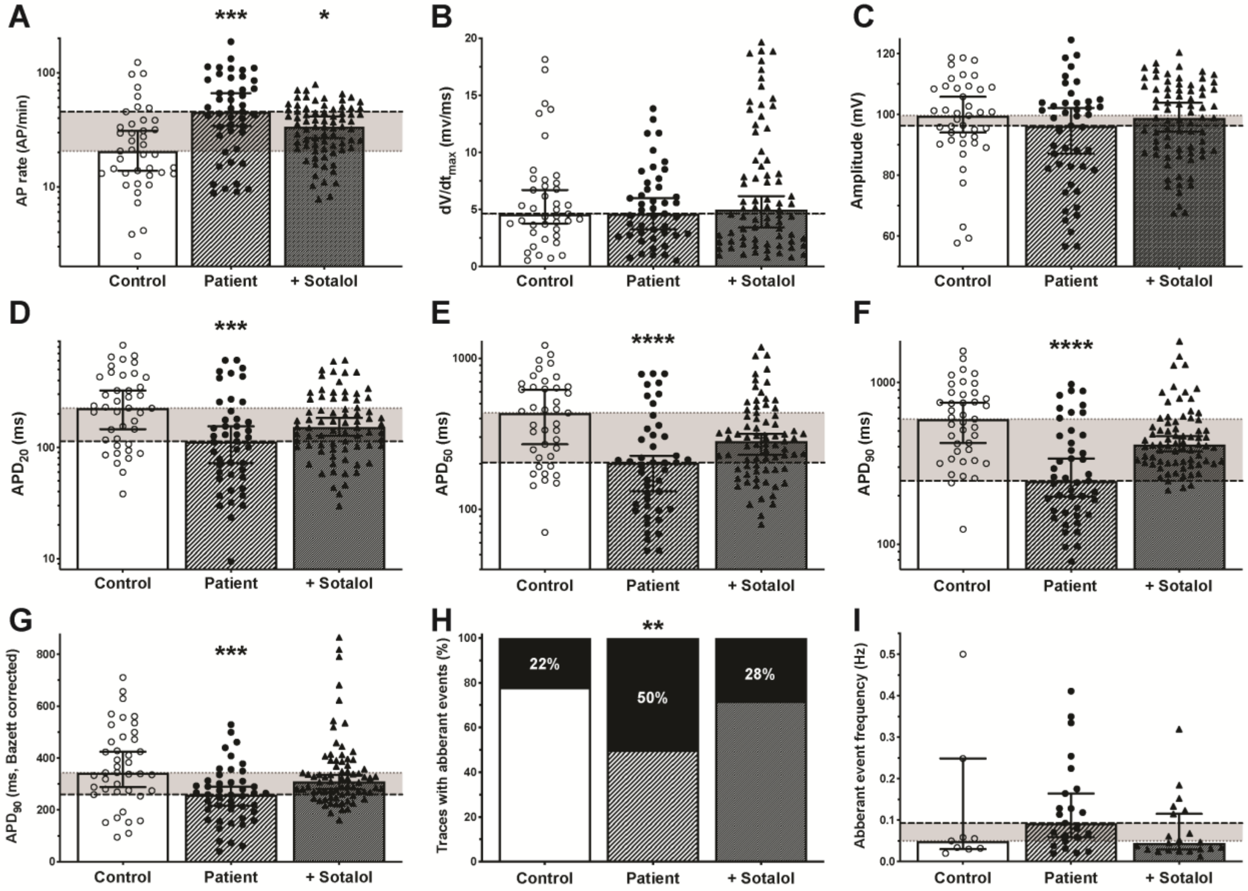

Supplementary Figure 8: Effect of Sotalol on spontaneous electrical activity
A-I. The AP properties of hiPSC-CM monolayers from control (n=41 cells) and patient specific without (n=45 cells) or with (n=75 cells) Sotalol were studied: AP rate (**A**), maximum depolarization slope (**B**), AP Amplitude (**C**), AP duration at 20% of repolarization (APD_{20}, **D**), APD_{50} (**E**), APD_{90} (**F**) and the APD_{90} corrected using the Bazett's formula (**G**), occurrence of aberrant electrical activity (control: 22% (9 out of 41); patient specific: 50% (24 out of 48); patient specific + sotalol: 28% (21 out of 75)) (**H**) and their frequency per recording (**I**). Histograms represent the median (95% confidence interval). The horizontal grey area illustrates the difference between the control and the patient specific conditions. *p<0.05, **p<0.01, ***p<0.001, ****p<0.0001 (Kruskal-Wallis test, except for H: Fisher's exact test).

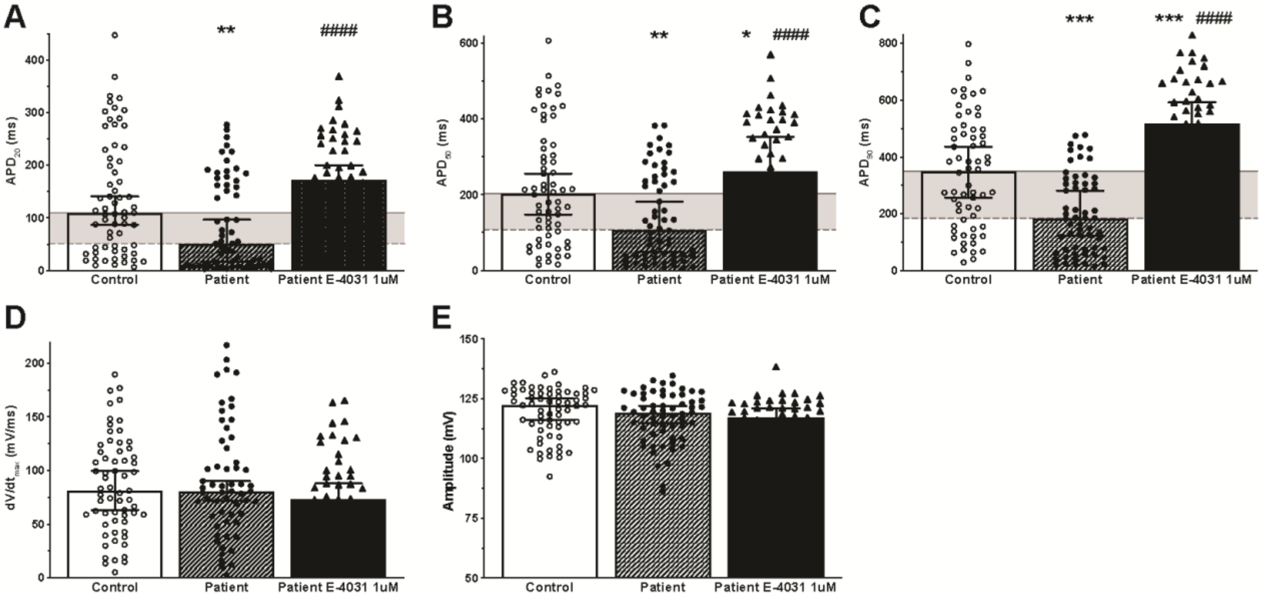

Supplementary Figure 9: Effect of E-4031 electrical activity evoked at 1Hz

A-E. AP parameters were evaluated for control (n=64), ACM patient specific (n=60) and ACM patient specific treated with 1µM of E-4031 hiPSC-CM: AP duration at 20% of repolarization (APD_{20}, **A**), APD_{50} (**B**), APD_{90} (**C**), maximum depolarization slope D), and AP Amplitude (**E**). ***** indicate differences with control condition, **#** indicate differences with patient condition. */# p<0.05, **/## p<0.01, ***/### p<0.001, ****/#### p<0.0001 (Kruskal-Wallis test).

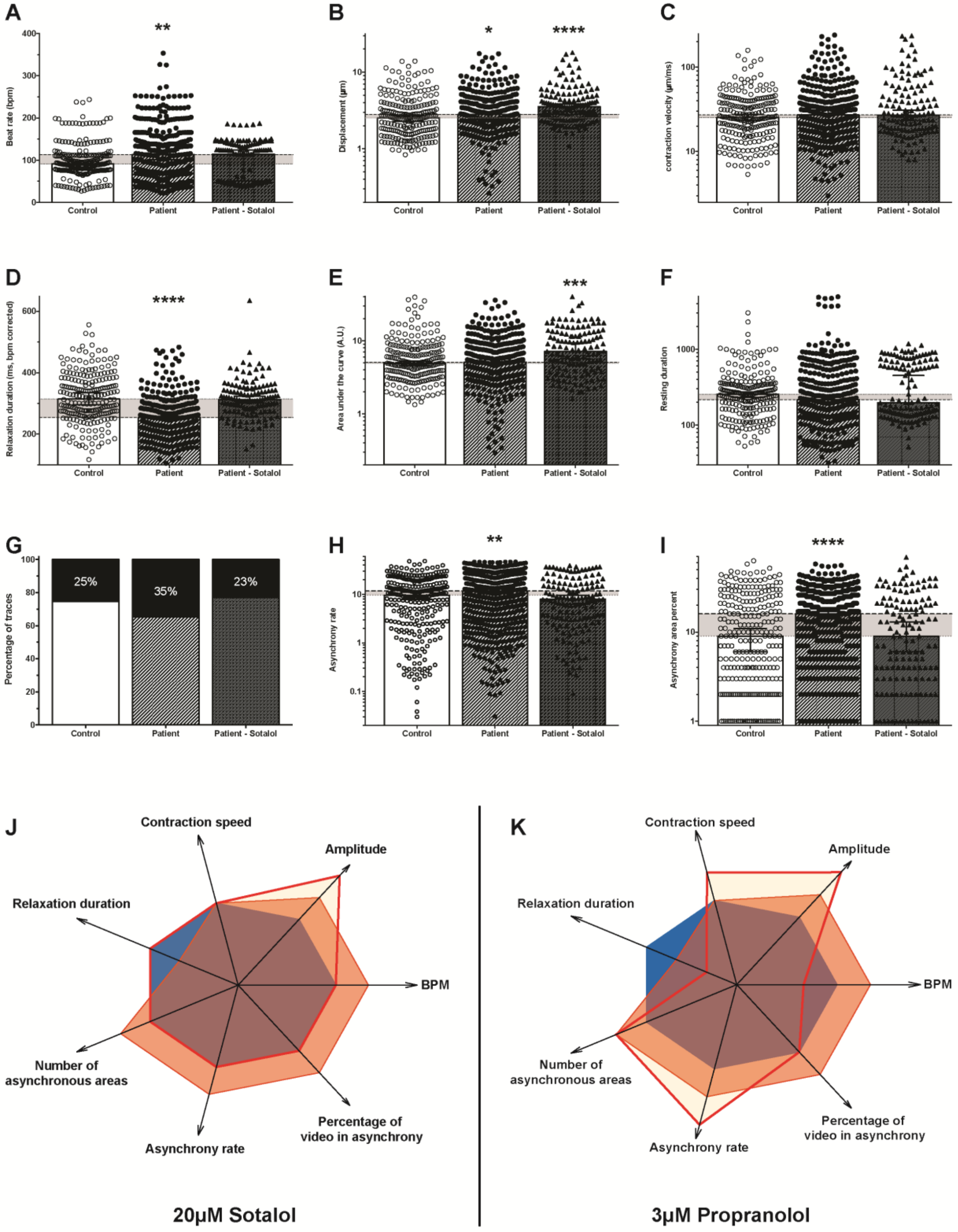

Supplementary Figure 10: Effect of Sotalol on spontaneous contractile activity

A-I. The contractile parameters of hiPSC-CM monolayers from control (n=229 videos) and patient specific without (n=359 videos) or with (n=128 videos) Sotalol were studied: the beat rate (**A**), the cellular displacement (**B**), the contraction velocity (**C**), the contraction duration from peak to 90% of relaxation corrected with Bazett's formula (**D**), the area under the curve (**E**), the resting duration (**F**), the percentage of videos demonstrating aberrant contractile events (control: 25% (278 areas out of 1097 total areas); patient specific: 35% (600 areas out of 1727 total areas); patient specific + sotalol: 23% (141 areas out of 614 total areas)) (**G**), the percentage of recording spent in asynchrony (**H**) and the percentage of the video area concerned by aberrant events (**I**). **J-K.** Spider chart is used to illustrate main differences observed between hiPSC-CM monomayres from control (blue area), patient specific without drugs (orange area) or patient specific with drugs (red line): sotalol (**J**) and propranolol (n=105 videos) (**K**). A "normal" value of 2 is attributed to the control parameter. Each evaluated parameter is compared to both the control and the patient specific conditions and fixed as higher or lower if statistically different.
Histograms represent the median (95% confidence interval). The horizontal grey area illustrates the difference between the control and the patient specific conditions. **p<0.01, ****p<0.0001 (Mann-Whitney test, except for (G): Fisher's exact test).

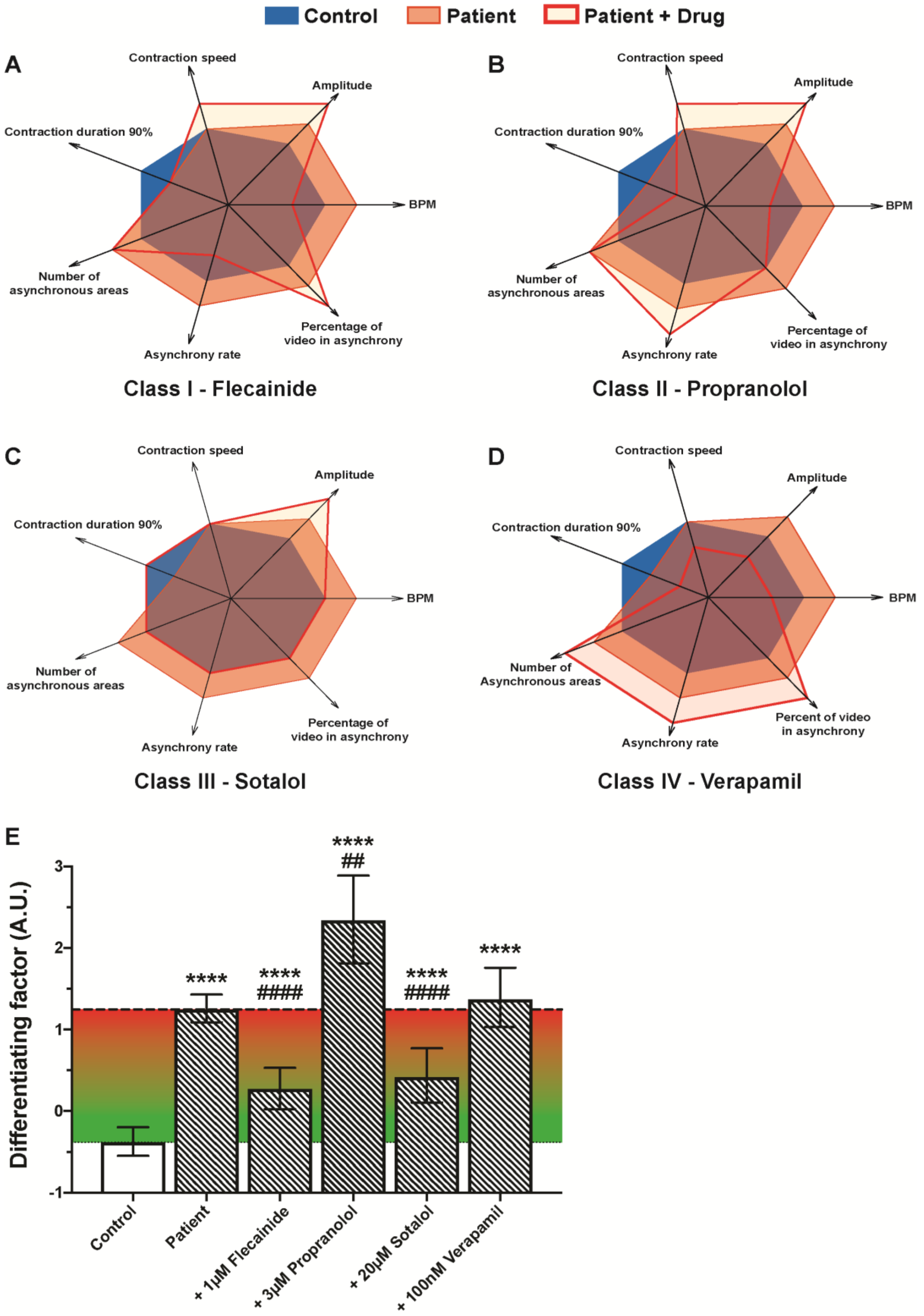

Control
Patient
Patient + Drug
A
Contraction speed
Amplitude
Contraction duration 90%
BPM
Number of asynchronous areas
Percentage of video in asynchrony
Asynchrony rate
Class I - Flecainide
B
Contraction speed
Amplitude
Contraction duration 90%
BPM
Number of asynchronous areas
Percentage of video in asynchrony
Asynchrony rate
Class II - Propranolol
C
Contraction speed
Amplitude
Contraction duration 90%
BPM
Number of asynchronous areas
Percentage of video in asynchrony
Asynchrony rate
Class III - Sotalol
D
Contraction speed
Amplitude
Contraction duration 90%
BPM
Number of Asynchronous areas
Percent of video in asynchrony
Asynchrony rate
Class IV - Verapamil
E
Differentiating factor (A.U.)
3
2
1
0
-1
****
****
####
****
##
****
####
****
Control
Patient
+ 1µM Flecainide
+ 3µM Propranolol
+ 20µM Sotalol
+ 100nM Verapamil

Supplementary Figure 11: AAD Evaluation using hiPSC-CM contractile properties
A-D. Spider chart is used to illustrate main differences observed between hiPSC-CM monolayers from control (blue area), patient specific without drugs (orange area) or patient specific with drugs (red line): 1µM flecainide (n=141 videos) (**A**), 3 µM propranolol (n=105 videos) (**B**), 20µM sotalol (n=128 videos) (**C**) and 100nM verapamil (n=85 videos) (**D**). A "normal" value of 2 is attributed to the control parameter. Each evaluated parameters are compared to both the control and the patient specific conditions and fixed as higher or lower if statistically different. **E.** Histogram depicting the "differentiating factor" obtained using a stepwise generalized linear model model (GLM). Once the linear model is identified, it is applied to the patient + treatment condition. Histograms represent the median (95% confidence interval). Differences were evaluated using a non-parametric Kruskal-Wallis test. ***** indicate differences with control condition, **#** indicate differences with patient condition. **/## p<0.01, ****/#### p<0.0001.

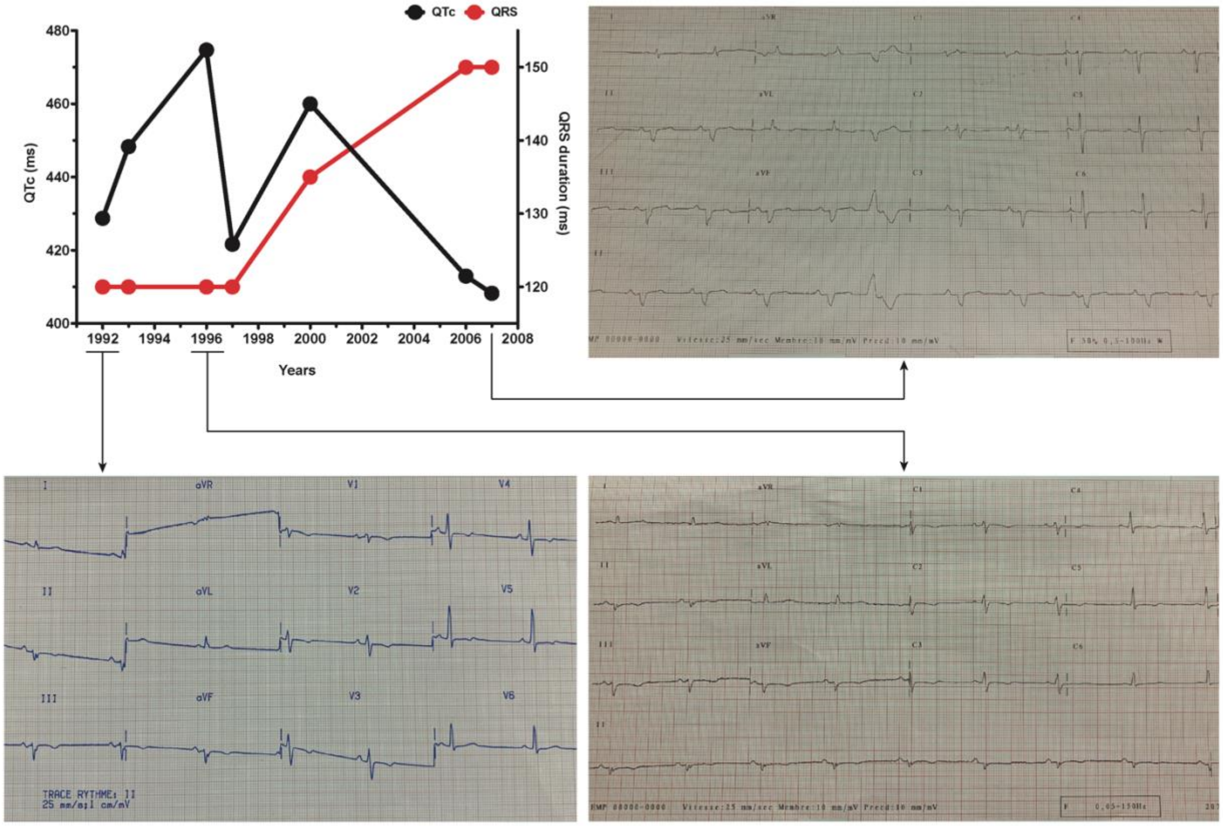

Supplementary Figure 12: Evolution of electrophysiological clinical parameters in the DSC2 - R132C ACM patient
Graph showing the evolution the QTc (black, left axis) and the QRS (red, right axis) duration of the index patient through a 16 years follow up (until the heart transplant procedure in 2007) (top left). Three surface ECG are shown for illustration.

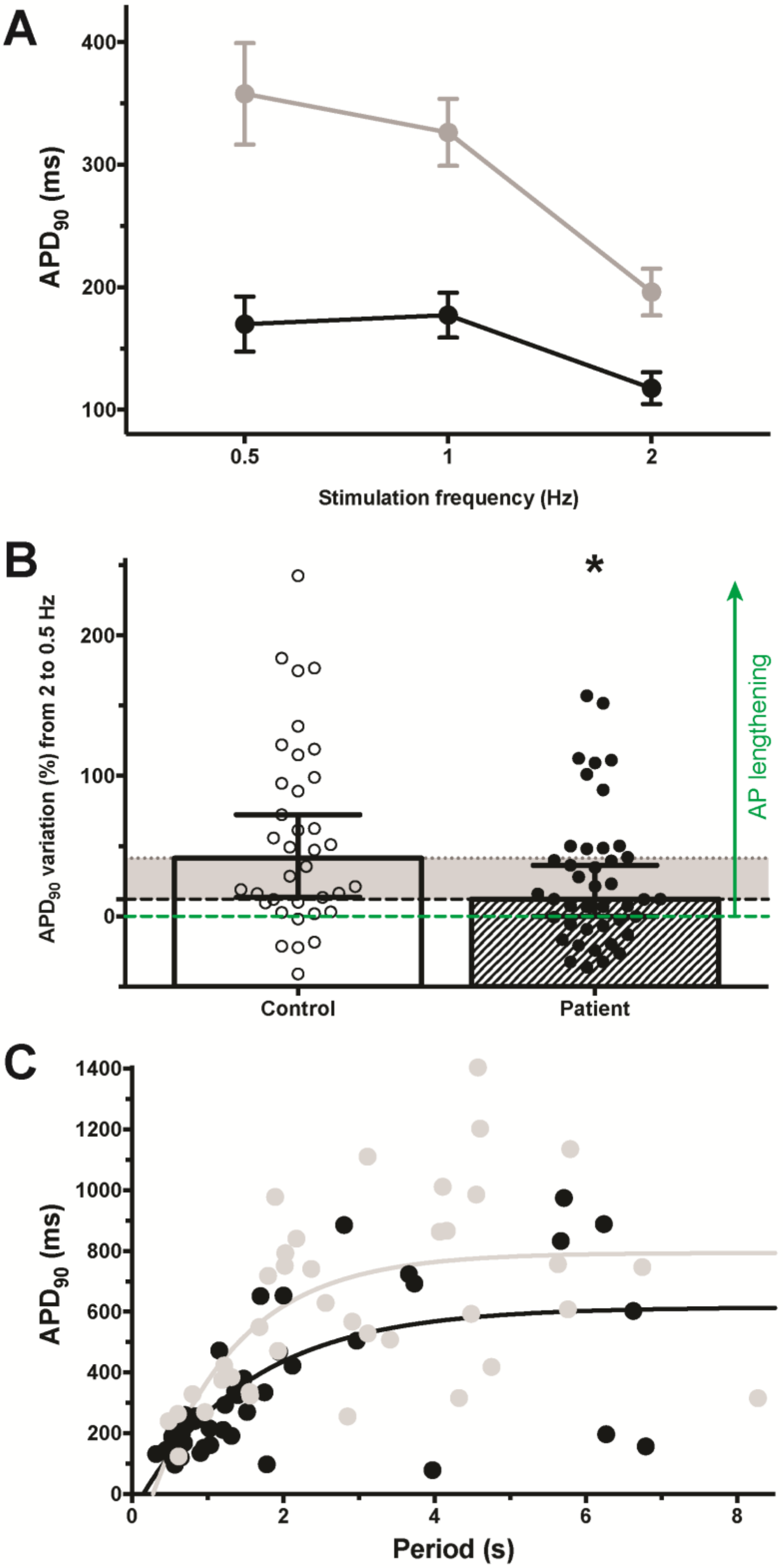

Supplementary Figure 13: APD90 frequency dependance of control and patient hiPSC-CM

A. Graph illustrating the APD_{90} for control (grey) and patient specific(black) hiPSC-CM paced at 0.5 (control n=45; patient n=53), 1 (control n=52 ; patient n=54) and 2 Hz (control n=36 ; patient n=43). **B**. For each complete recording (control n=36; patient n=42), the APD_{90} variation percentage from AP paced at 2 Hz to AP paced at 0.5 Hz is shown. Values greater than 0 indicate an AP lengthening when cells are paced from 2 to 0.5 Hz. **C**. Regarding the spontaneous electrical activity, the APD_{90} is plotted as a function of the mean period between 2 spontaneous AP during each recording, as an indication of the APD_{90} dependency to the spontaneous AP rate.

Supplementary movie 1: The beating heart of a 3dpf wild-type embryo
High-speed video recording (120 fps) capturing the beating heart of a 3dpf wild-type embryo.

Supplementary movie 2: The beating heart of a 3dpf dsc2lATG-morphant
High-speed video recording (120 fps) capturing the beating heart of a dsc2l ATG-morphant.

Supplementary movie 3: The beating heart of a 3dpf dsc2lsplice-morphant
High-speed video recording (120 fps) capturing the beating heart of a dsc2l splice-morphant.

Supplementary movie 4: Spontaneously beating control hiPSC-CM
Video recording (30 fps) of spontaneously beating 60 days old control hiPSC-CM monolayer.

Supplementary movie 5: Spontaneously beating patient specific hiPSC-CM
Video recording (30 fps) of spontaneously beating 60 days old patient specific hiPSC-CM monolayer.
